# Synchronization of interconnected heterogeneous networks: The role of network sizes

**DOI:** 10.1038/s41598-019-42636-6

**Published:** 2019-04-16

**Authors:** Huixin Zhang, Weidong Zhang, Jianxi Gao

**Affiliations:** 1 Shanghai Jiao Tong University, Automation, Shanghai, 200240 China; 2 Rensselaer Polytechnic Institute, Computer Science Department & Network Science and Technology Center, Troy, New York, 12180 USA

## Abstract

Increasing evidence shows that real networks interact with each other, forming a network of networks (NONs). Synchronization, a ubiquitous process in natural and engineering systems, has fascinatingly gained rising attentions in the context of NONs. Despite efforts to study the synchronization of NONs, it is still a challenge to understand how do the network sizes affect the synchronization and its phase diagram of NONs coupled with nonlinear dynamics. Here, we model such NONs as star-like motifs to analytically derive the critical values of both the internal and the external coupling strengths, at which a phase transition from synchronization to incoherence occurs. Our results show that the critical values strongly depend on the network sizes. Reducing the difference between network sizes will enhance the synchronization of the whole system, which indicates the irrationality of previous studies that assume the network sizes to be the same. The optimal connection strategy also changes as the network sizes change, a discovery contradicting to the previous conclusion that connecting the high-degree nodes of each network is always the most effective strategy to achieve synchronization unchangeably. This finding emphasizes the crucial role of network sizes which has been neglected in the previous studies and could contribute to the design of a global synchronized system.

## Introduction

The present high attention to network science roots in its capability of explaining the behavior of complex real systems, including Internet^1,2^, sociology^3^, biology^4^, transportation^5^, and power grids^6^. For these real-world systems, one component is often an individual network within a much larger complex multilayer network^7–10^, forming a part of a network of networks (NON). Comparing to a single network, the connection between networks brings more complex and complicated properties of NONs, such as robustness^8,11–13^, epidemic eruption^14^, and the self-organization in evolutionary game^15^. Among these properties, synchronization^16^ is an important behavior in biological^17^, engineering^18^, climatological^19^, and ecological systems^20^. The synchronization of a single network has been intensively investigated in the past few decades. However, the synchronization of NONs, which is more universal in real world, has not gained enough understanding^21^ yet. There has been a limited number of studies on the synchronization of NONs. Martin *et al*.^22^ showed the existence of a critical number of diagonal interlinks, beyond which synchronization could not be enhanced, while Francesco^23^ introduced a unique Master Stability Function that determines the stability of synchronization. The study on the synchronization of NONs has certain practical significance. For instance, the smart-grid, which can be regarded as NONs^24^, is a combination of microgrids and the traditional power networks. More and more microgrids are integrated into the power system, consequently decreasing the couplings of the power system and thus driving this NON into the boundary of its synchronization^18^.

Three key factors dominate the synchronization of NONs: the connection strategy, *i*.*e*. the linkage between different networks; the dynamics, *i*.*e*. the universally nonlinear behavior in the real world; and the structure, *i*.*e*. the connections between a certain amount of nodes. While research on these three factors have led to some important findings, some scientific gaps remain. Some studies found that the optimal connection strategy for the case of a single connection is always to link the high-degree nodes from different networks^25,26^. No model, to our best knowledge, are available to systematically study and theoretically explain the optimal connection strategies for more than one connection. Previously, the most commonly applied predictive tools to study the dynamics of NONs, such as Master Stability Function^27^ and the eigenvalues of the adjacency matrix^18,28^, are based on the linearization (*e*.*g*. replacing $\sin \,({\theta }_{i}-{\theta }_{j})$ by ${\theta }_{i}-{\theta }_{j}$). A more accurate model should have the ability to utilize the original nonlinear dynamics to provide a more accurate prediction. Moreover, these tools introduce the usage of super-Laplacian matrix^29^ for NONs, requiring huge time for calculations. In the previous studies, the network sizes, defined as the number of nodes, between different networks are assumed the same^30–34^. But in the real-world systems, the sizes between different networks are usually different and may change over time. One example is the smart-grid^24^. The number of the generating units of the grid-connected microgrids is significantly different from that of the traditional power grid, and the former number might vary by time due to unstable resources. Another example is the plant-animal mutualistic network^35^. In the mutualistic ecological networks, the numbers of species are different between plant and animal, and species’ numbers of both plant and animal are changing due to the extinction and evolution. When the network sizes change, the results derived by these current tools need to be updated^18,27,28,36^ since they require the exact adjacency matrix, which could change dramatically even with a tiny change in network sizes. This property determines that for NONs with fast-changeable sizes, these tools have to recalculate frequently every time the network size changes, causing low performance in time-demanding control. Therefore, a new mathematical tool is in need for the fast-changing network sizes in NONs.

In this paper, we propose a mathematical framework to study the synchronization of NONs, with nonlinear dynamics and fast-changing network sizes. Firstly, for different connection strategies, we observe that the decrease in coupling strengths or the increase of network sizes may drive a synchronized system to be unsynchronized. Secondly, fixing network sizes, we derive the analytical expressions of the critical coupling strengths, at which a phases transition from synchronization to incoherence occurs. In return, fixing the coupling strengths, we can mathematically provide the thresholds of allowable network sizes, above which one system could not keep synchronized. This finding has important implications. In the case of modern power grid, our study can provide the maximum allowable number of microgrids that the power system can tolerate. Thirdly, we give the phase diagram of the allowable network sizes, guiding how to adjust the coupling strengths for keeping system synchronized with the fast-changing network sizes. Finally, the phase diagrams for different connection strategies are compared to find the optimal one. In contrast to previous findings, we show that to connect the nodes with high degrees is not always the best connection strategy; the optimal strategy depends on the network sizes. Our results light up the important role of network sizes and may help to design a global synchronized system.

## Results

### The Model

**Figure 1:**
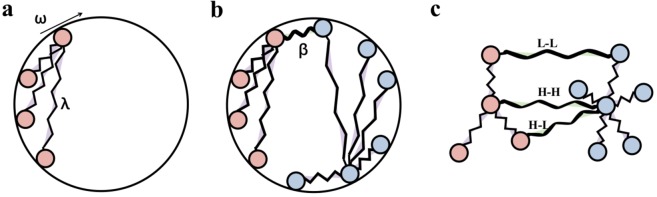
The mechanical analog of the oscillator-based networks. (**a**) In one single oscillator-based network, synchronized oscillators with the intrinsic rotation frequency $\omega$ are coupled through the elastic spring *λ* to simultaneously rotate around the circle without colliding. (**b**) With interdependence, two oscillator-based networks (red color present one network, while blue color present the other network) share another kind of elastic spring *β* to rotate around the circle together. (**c**) These two networks can be coupled by 3 connection strategies, including hub connecting to hub (**H**-**H**), hub connecting to leaf (**H**-**L**), and leaf connecting to leaf (**L**-**L**).

One of the most widely studied models of synchronization is the Kuramoto model^37^ (see Method Kuramoto Model). We present a mechanical analog of this model in Fig. 1a, where nodes are coupled through the coupling strength *λ*. The nodes are synchronized if they rotate around a circle with angular velocity ω and avoid collisions. In the single network Kuramoto model, the system undergoes a first order^38^ or a hysteresis transition^39^ when the coupling strength crosses a critical value. Comparing to a single network, NONs behaves much more complex since it has two kinds of coupling strengths: the internal coupling strength *λ* and the external coupling strength *β* (see Fig. 1b and Method Coupled Kuramoto Model). As seen in Fig. 1c, previous studies^25,26^ raised three basic forms of connection strategies: the hub connects to the hub (**H**-**H**), the hub connects to the leaf (**H**-**L**) and the leaf connects to the leaf (**L**-**L**). Here, we not only consider the connection strategy with one link but also with two links (see Method Connection Strategy).

**Figure 2:**
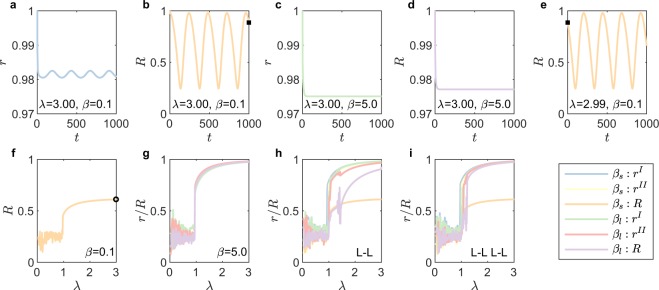
Phase transition with different coupling strengths. Two networks with ${N}^{I}=30$ and ${N}^{II}=50$ are coupled through connection strategies **H**-**H** (subplots (**a**–**f**)), **L**-**L** (subplot (**h**)) and **L**-**L L**-**L** (subplot **i**). (**a**) shows that *r*^*I*^(*t*), the order parameter of Network I, is a periodic function of time *t* with the coupling strengths $\lambda =3.00$, $\beta =0.1$ (small value of *β*, *i*.*e*., *β*_*s*_ in the legend). (**b**) Indicates that *R*(*t*), the order parameter of the whole system, is also a periodic function of *t* with $\lambda =3.00$, $\beta =0.1$. (**c** and **d**) Show when $\lambda =3.00$, $\beta =5.0$ (large value of *β*, i.e. *β*_*l*_ in the legend), *r*^*I*^(*t*) and *R*(*t*) converge to their steady states. In subplots (**a**–**d**), the initial phases of all oscillators are 1. In subplot (**e**), the initial state of the phases ${\theta }_{i}(t=0,\lambda -{\rm{\Delta }}\lambda )$ is equal to the final state of the phase ${\theta }_{i}(t=0,\lambda )$ (**b**), with $\lambda =3.00$ and ${\rm{\Delta }}\lambda =0.01$ as indicated by ■. (**f**) Shows that how *R*, the global order parameter, changes as *λ* changes. $\circ$ presents the value of *R* with $\lambda =2.99$, $\beta =0.1$, which is the mean value during one period in (**e**). With small *R*, the system is aways unsynchronized with ${\beta }_{s}=0.1$. In (**g**), with ${\beta }_{l}=5.0$ the system is globally synchronized for large *λ* and has a phase transition from synchronization to incoherency. Subplots (**h**) (**L**-**L**) and **i** (**L**-**L L**-**L**) show order parameters as a function of *λ*, with both ${\beta }_{s}=0.1$ and ${\beta }_{l}=5.0$.

In this paper, we mainly focus on the case of a NON composed of two networks, which can be easily extended to the case of multiple networks by introducing new network layers and the connections between the networks. The order parameter to quantify the synchronization of a single network^37^ is $r(t){e}^{{\rm{i}}\varphi }=\frac{1}{N}({\sum }_{j=1}^{N}\,{e}^{{\rm{i}}{\theta }_{{\rm{j}}}({\rm{t}})})$, where *r*(*t*) is the order parameter at time step *t*, *N* is the network size, *θ*(*t*) is the phase at time *t*, and i denotes an imaginary number. The order parameters for network I and network II are set as *r*^*I*^(*t*) and *r*^*II*^(*t*). The upper indices (I) and (II) stand for ‘Network I’ and ‘Network II’ respectively. Furthermore, we define the order parameter for interconnected networks *R*(*t*) as:

1 $$R(t){e}^{{\rm{i}}\varphi }=\frac{1}{{N}^{I}+{N}^{II}}(\sum _{j=1}^{{N}^{I}}\,{e}^{i{\theta }_{j}^{I}(t)}+\sum _{j=1}^{{N}^{II}}\,{e}^{{\rm{i}}{\theta }_{{\rm{j}}}^{{\rm{II}}}({\rm{t}})}),$$

where $\varphi$ is the average phase of the both networks. Thus, we can generate two Scale-Free (SF) networks (see Method Scale-Free Networks) with ${N}^{I}=30$ and ${N}^{II}=50$, interconnected by the **H**-**H** strategy. Then numerically mimic the order parameter *r*(*t*) by solving the ordinary differential equations of the coupled Kuramoto model (See Method Coupled Kuramoto model) for the given internal and external coupling strengths, where $t\in [0,T]$. As shown in Fig. 2a,b, when $\lambda =3$ and $\beta =0.1$ (small value of *β*, *i*.*e*. *β*_*s*_ in the legend) both order parameters *r*^*I*^(*t*) and *R*(*t*) show a periodic function as time *t* and we denote the period as Δ*T* (Case I), indicating that the interconnected networks are not globally synchronized. When $\lambda =3$ and $\beta =5$ (large value, *i*.*e*. *β*_*l*_ in the legend), however, both *r*^*I*^(*t*) and *R*(*t*) converge to their steady states (Case II), demonstrating that the interconnected networks are in the globally synchronized state, showing in Fig. 2c,d. To systematically study how the internal coupling strength, *λ*, determines the synchronization of interconnected networks, we decrease *λ* from 3 to 0 gradually with a constant interval ${\rm{\Delta }}\lambda =0.01$. Furthermore, the initial state of the phases ${\theta }_{i}(t=0,\lambda -{\rm{\Delta }}\lambda )$ is equal to the final state of the phase ${\theta }_{i}(t=0,\lambda )$, as shown in Fig. 2b,e. Next, we define the order parameter for the given *λ* at the final state as

2 $$R=\{\begin{array}{cc}\langle R(t)\rangle {|}_{t\in [T-{\rm{\Delta }}T,T]}, & {\rm{C}}{\rm{a}}{\rm{s}}{\rm{e}}\,{\rm{I}}\\ R(T), & {\rm{C}}{\rm{a}}{\rm{s}}{\rm{e}}\,{\rm{I}}{\rm{I}}\end{array},$$

and the definition of *r* is similar to equation (2) by replacing *R* to *r*. The global order parameter *R* is a decreasing function of *λ* as shown in Fig. 2f, demonstrating that the system is always in the unsynchronized state for small $\beta =0.1$. When *β* is large (Fig. 2g), the system is globally synchronized for large *λ* and shows a phase transition from synchronization to incoherency when *λ* crosses a critical value *λ*_*c*_. Similarly, we also investigate the order parameters *r* and *R* as function of *λ* for two interconnected SF networks with **L**-**L** (Fig. 2h) and **L**-**L**, **L**-**L** strategies (Fig. 2i). We find that the system with a small value of the external coupling strength *β*_*s*_ can never synchronize, *i*.*e*., it is independent of the value of the internal coupling strength *λ* (in yellow). Meanwhile, for the large value *β*_*l*_, there exists the critical value of *λ* that separates the synchronized phase and the unsynchronized phase, indicating the existence of a minimum value of *β*, below which the system can never synchronize (more details in Supplementary Sec. S2). To study the critical coupling strength of SF networks^2^, one common method is analyzing the star-like networks^40,41^, a close proxy when the SF network has sufficient heterogeneity^42^. Thus, we develop an analytical method to study the critical internal and external coupling strengths with star-like motifs and validate our approach on interconnected SF networks.

### The Synchronization conditions

Without loss of generality, the network with more nodes is set as Network I, while the network with smaller size is defined as Networks II, *i*.*e*., ${N}^{I}\ge {N}^{II}$ (for the derived process). When the two interconnected networks are synchronized, the phase differences between nodes would no-longer change, defined as locking manifolds^40^. Using this condition, we obtain the critical values as below (the derived process can be found in Supplementary Sec. S3 and S4):

3 $${\lambda }_{c}^{{\bf{o}}{\bf{n}}{\bf{e}} \mbox{-} {\bf{l}}{\bf{i}}{\bf{n}}{\bf{k}}}=\{\begin{array}{cc}\frac{{N}^{I}+{N}^{II}-4}{{N}^{I}+{N}^{II}}\omega , & {H}^{I}\in C\\ \frac{3{N}^{I}-{N}^{II}-4}{{N}^{I}+{N}^{II}}\omega , & {H}^{I}\notin C\end{array},$$

where *H*^*I*^ stands for the hub in Network I and *C* is the set of nodes linking both networks;

4 $${\beta }_{c}^{{\bf{o}}{\bf{n}}{\bf{e}}{\boldsymbol{ \mbox{-} }}{\bf{l}}{\bf{i}}{\bf{n}}{\bf{k}}}=2\frac{{N}^{I}-{N}^{II}}{{N}^{I}+{N}^{II}}\omega .$$

Here, **one**-**link** includes all the four connection strategies: **H**-**H**, **H**-**L**, **L**-**H** and **L**-**L**.

Next, we derive the analytical solutions for the case of two-links connection. The analytical critical internal coupling strength can be mathematically described as

5 $${\lambda }_{c}^{{\bf{t}}{\bf{w}}{\bf{o}} \mbox{-} {\bf{l}}{\bf{i}}{\bf{n}}{\bf{k}}{\bf{s}}}=\{\begin{array}{cc}\begin{array}{c}{\xi }_{2},\\ max\,\{{\xi }_{2},\,min\,\{2{\xi }_{3},2{\xi }_{2}+2{\xi }_{3}-2\sqrt{2{\xi }_{2}{\xi }_{3}}\}\},\\ 2{\xi }_{1},\end{array} & \begin{array}{cc}{H}^{I}\in C & {\rm{\& }}{H}^{II}\in C\\ {H}^{I}\in C & {\rm{\& }}{H}^{II}\notin C\\ {H}^{I}\notin C & \end{array}\end{array},$$

while the critical external coupling strengths are

6 $$\begin{array}{ccc}{\beta }_{c}^{{\bf{H}} \mbox{-} {\bf{H}}\,{\bf{L}} \mbox{-} {\bf{L}}} & = & max\,\{\frac{-\,2\lambda +4{\xi }_{1}}{\frac{\pi }{2}+\arcsin \,(1-\frac{2{\xi }_{2}}{\lambda })},-\frac{\pi +2}{8}\lambda +\,{\xi }_{3}+\sqrt{{(\frac{\pi +2}{8}\lambda -{\xi }_{3})}^{2}+\lambda {\xi }_{3}}\},\end{array}$$

7 $$\begin{array}{ccc}{\beta }_{c}^{{\bf{H}} \mbox{-} {\bf{L}}\,{\bf{L}} \mbox{-} {\bf{H}}} & = & max\,\{\frac{-\,2\lambda +4{\xi }_{1}}{\frac{\pi }{2}+\arcsin \,(1-\frac{2{\xi }_{3}}{\lambda })},-\frac{\pi +2}{8}\lambda +\,{\xi }_{1}+\sqrt{{(\frac{\pi +2}{8}\lambda -{\xi }_{1})}^{2}+\lambda {\xi }_{3}}\},\end{array}$$

8 $$\begin{array}{ccc}{\beta }_{c}^{{\bf{H}} \mbox{-} {\bf{L}}\,{\bf{L}} \mbox{-} {\bf{L}}} & = & max\,\{\frac{-\,2\lambda +4{\xi }_{1}}{\frac{\pi }{2}+\arcsin \,(1-\frac{2{\xi }_{2}}{\lambda })+\arcsin \,(1-\frac{2{\xi }_{3}}{\lambda })},-\frac{\pi +2}{12}\lambda +\frac{2{\xi }_{1}+3{\xi }_{3}}{6}+\sqrt{{(\frac{\pi +2}{12}\lambda -\frac{2{\xi }_{1}+3{\xi }_{3}}{6})}^{2}+\frac{2}{3}\lambda {\xi }_{3}}\},\end{array}$$

9 $${\beta }_{c}^{{\bf{L}}{\boldsymbol{ \mbox{-} }}{\bf{H}}\,{\bf{L}}{\boldsymbol{ \mbox{-} }}{\bf{L}}}=-\,\frac{\pi +2}{12}\lambda +\frac{-\,{\xi }_{3}+2{\xi }_{4}}{6}+\sqrt{{(\frac{\pi +2}{12}\lambda -\frac{-{\xi }_{3}+2{\xi }_{4}}{6})}^{2}-\frac{2}{3}\lambda {\xi }_{3}},$$

10 $${\beta }_{c}^{{\bf{L}}{\boldsymbol{ \mbox{-} }}{\bf{L}}\,{\bf{L}}{\boldsymbol{ \mbox{-} }}{\bf{L}}}=\frac{{N}^{I}-{N}^{II}}{{N}^{I}+{N}^{II}}\omega ,$$

where ${\xi }_{1}=\frac{{N}^{I}-2}{{N}^{I}+{N}^{II}}\omega$, ${\xi }_{2}=\frac{{N}^{I}+{N}^{II}-4}{{N}^{I}+{N}^{II}}\omega$, ${\xi }_{3}=\frac{{N}^{I}-{N}^{II}}{{N}^{I}+{N}^{II}}\omega$, ${\xi }_{4}=\frac{{N}^{II}-2}{{N}^{I}+{N}^{II}}\omega$. Different from one-link connection, ${\beta }_{c}^{two \mbox{-} links}$ depends on the value of *λ* except **L**-**L L**-**L**. Note that when $\lambda  > {\lambda }_{c}$ and $\beta  > {\beta }_{c}$ the two networks are guaranteed to be synchronized together for **H**-**H L**-**L**, **H**-**L L**-**H** and **L**-**L L**-**L** connection strategies. It is, however, not a sufficient condition for both **H**-**L L**-**L** and **L**-**H L**-**L** connection strategies. If only one hub acts as a connector (**H**-**L L**-**L** and **L**-**H L**-**L**), only when $2{\xi }_{4} < \lambda  < 2{\xi }_{2}+4{\xi }_{4}-4\sqrt{{\xi }_{2}{\xi }_{4}}$ and $\beta \in [{\beta }_{c},{\beta }_{{\rm{\max }}}]$, the whole system is synchronized, where the maximum external coupling strength satisfies

11 $${\beta }_{{\rm{\max }}}^{{\bf{H}}{\boldsymbol{ \mbox{-} }}{\bf{L}}\,{\bf{L}}{\boldsymbol{ \mbox{-} }}{\bf{L}},{\bf{L}}{\boldsymbol{ \mbox{-} }}{\bf{H}}\,{\bf{L}}{\boldsymbol{ \mbox{-} }}{\bf{L}}}=\frac{-\,2\lambda +4{\xi }_{4}}{\frac{\pi }{2}+\arcsin \,(1-\frac{2{\xi }_{2}}{\lambda })+\arcsin \,(1-\frac{4{\xi }_{4}}{\lambda })}.$$

It means that in this case the system not only has the lower limit *β*_*c*_ but also has the upper limit *β*_max_.

**Figure 3:**
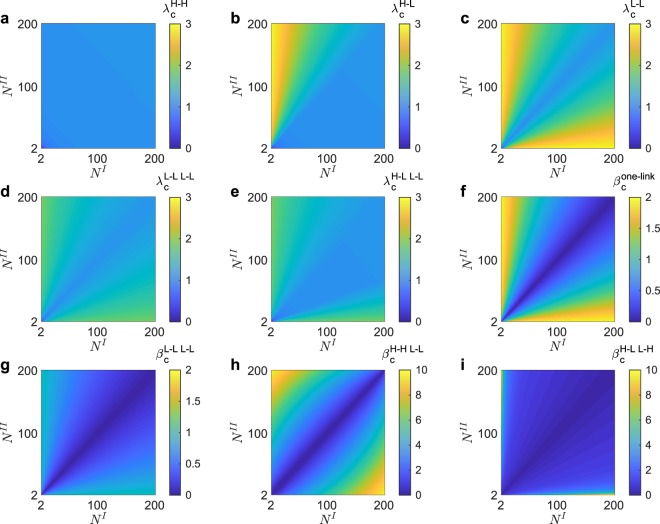
The critical coupling strengths. In each subplot, the color stands for the derived values of critical coupling strengths, as a function of networks sizes *N*^*I*^ and *N*^*II*^. (**a**) The derived results of *λ*_*c*_ for **H**-**H** (equation (3) for case ${H}^{I}\in C$), as well as **H**-**H L**-**L** and **H**-**L L**-**H** (equation (5) for case ${H}^{I}\in C\& {H}^{II}\in C$). (**b**) ${\lambda }_{c}^{{\bf{H}}{\boldsymbol{ \mbox{-} }}{\bf{L}}}$, combing with the lower triangle area (equation (3) for case ${H}^{I}\in C$) and the upper triangle area (equation (3) for case ${H}^{I}\notin C$). (**c**) ${\lambda }_{c}^{{\bf{L}}{\boldsymbol{ \mbox{-} }}{\bf{L}}}$ (equation (3) for case ${H}^{I}\notin C$). (**d**) The derived ${\lambda }_{c}^{{\bf{L}}{\boldsymbol{ \mbox{-} }}{\bf{L}}\,{\bf{L}}{\boldsymbol{ \mbox{-} }}{\bf{L}}}$ (equation (5) for ${H}^{I}\notin C$). (**e**) ${\lambda }_{c}^{{\bf{H}}{\boldsymbol{ \mbox{-} }}{\bf{L}}\,{\bf{L}}{\boldsymbol{ \mbox{-} }}{\bf{L}}}$ is combined with the lower triangle area (equation (5) for case ${H}^{I}\in C\& {H}^{II}\notin C$) and the upper triangle area (equation (5) for case ${H}^{I}\notin C$). (**f**) ${\beta }_{c}^{{\bf{o}}{\bf{n}}{\bf{e}}{\boldsymbol{ \mbox{-} }}{\bf{l}}{\bf{i}}{\bf{n}}{\bf{k}}}$ (4). (**g**) ${\beta }_{c}^{{\bf{L}}{\boldsymbol{ \mbox{-} }}{\bf{L}}\,{\bf{L}}{\boldsymbol{ \mbox{-} }}{\bf{L}}}$ (equation (10)). (**h**) ${\beta }_{c}^{{\bf{H}}{\boldsymbol{ \mbox{-} }}{\bf{H}}\,{\bf{L}}{\boldsymbol{ \mbox{-} }}{\bf{L}}}$ (equation (6)). (**i**) ${\beta }_{c}^{{\bf{H}}{\boldsymbol{ \mbox{-} }}{\bf{L}}\,{\bf{L}}{\boldsymbol{ \mbox{-} }}{\bf{H}}}$ (equation (7)).

**Figure 4:**
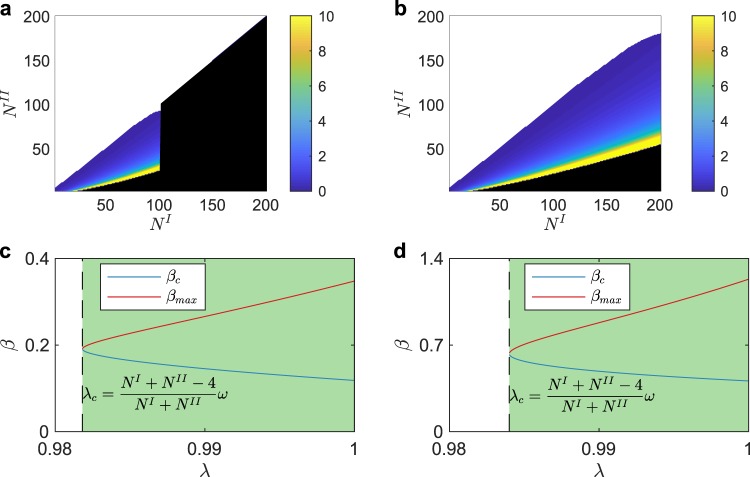
The lower and the upper limit of *β* in **H**-**L L**-**L**. Different from other connection strategies, *β*^**H**-**L L**-**L**^ has the upper limit *β*_*max*_ as well as the lower limit *β*_*c*_. In (**a** and **b**), if the upper limit is less than the lower limit, *i*.*e*., ${\beta }_{c} > {\beta }_{max}$, the system could not synchronize and we color the corresponding points as white. Meanwhile, *β*_*max*_ limits *λ*, which means when *λ* is out of a certain range the system could not synchronize. If $\lambda$ (λ = 0.98 in (**a**), λ = 0.99 in (**b**)) does not belong to such range, the corresponding points in (**a** and **b**) are colored as black. (**c** and **d**) Show how *β*_*c*_ and *β*_*max*_ change as *λ*, for certain network sizes. (**c**) ${N}^{I}=120$, ${N}^{II}=100$, ${\lambda }_{c}=0.9818$. (**d**) ${N}^{I}=150$, ${N}^{II}=100$, ${\lambda }_{c}=0.9840$.

In the rest of this article, we do not assume ${N}^{I}\geqslant {N}^{II}$ and thus **H**-**L** equals to **L**-**H**. Figure 3 shows the derived results of critical coupling strengths, as indicated in equations (3–10). In Fig. 3, the color of each point (*N*^*I*^, *N*^*II*^) presents the critical coupling strength with certain networks sizes *N*^*I*^ and *N*^*II*^. Figure 3a demonstrates ${\lambda }_{c}=\frac{{N}^{I}+{N}^{II}-4}{{N}^{I}+{N}^{II}}\omega$, which is the critical internal coupling strength for the cases **H**-**H**, **H**-**H L**-**L**, and **H**-**L L**-**H**. This *λ*_*c*_ increases with ${N}^{I}+{N}^{II}$, indicating that a larger system displays less synchronization, which coincides with experimental studies in power systems^43^ and is analogous to the discovery in etiological systems^44^. Figure 3b–e show *λ*_*c*_ for **H**-**L**, **L**-**L**, **L**-**L L**-**L** and **H**-**L L**-**L** respectively. We observe that Fig. 3b (**H**-**L**) is the combination of Fig. 3a (**H**-**H**) and Fig. 3c (**L**-**L**), along the diagonal line ${N}^{I}={N}^{II}$. By comparing Figs 3e to 3d,c, we found that the upper triangle of case **H**-**L L**-**L**, *i*.*e*., ${N}^{I}\leqslant {N}^{II}$, is as the same as that of case **L**-**L L**-**L**. Such property depends on whether the hub with larger network size acts as the connector (*H*^*I*^ in equations (2) and (4)), indicating the important role of network size in deciding the critical internal coupling strength. Figure 3f–i show *β*_*c*_ for **one**-**link**, **L**-**L L**-**L**, **H**-**H L**-**L** and **H**-**L L**-**H** respectively. These critical external couplings increase with $|{N}^{I}-{N}^{II}|$ and decrease with ${N}^{I}+{N}^{II}$, indicating the importance of absolute network sizes ${N}^{I}+{N}^{II}$ and the relative network sizes $|{N}^{I}-{N}^{II}|$. Figure 4 is about *β*^**H**-**L L**-**L**^, which has the upper limit *β*_*max*_ as well as the lower limit *β*_*c*_. If the upper limit is less than the lower limit, *i*.*e*., ${\beta }_{c} > {\beta }_{{\max }}$, the system could not synchronize and we set the color of the corresponding points in Fig. 4a and b white. Moreover, both *β*_*c*_ and *β*_*max*_ are closely related to *λ*, requiring *λ* belongs to a certain range (Eqs (8), (9) and (11)). If $\lambda =0.98$ ($\lambda =0.99$) does not belong to such certain range, the corresponding points in Fig. 4a,b are colored as black. In Fig. 4c and d, we show how ${\beta }_{c}^{{\bf{H}}{\boldsymbol{ \mbox{-} }}{\bf{L}}\,{\bf{L}}{\boldsymbol{ \mbox{-} }}{\bf{L}}}$ and ${\beta }_{{\max }}^{{\bf{H}}{\boldsymbol{ \mbox{-} }}{\bf{L}}\,{\bf{L}}{\boldsymbol{ \mbox{-} }}{\bf{L}}}$ are closely related to *λ*.

**Figure 5:**
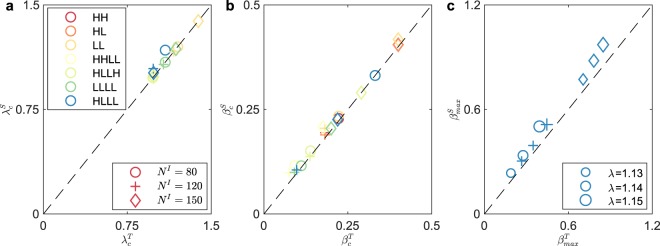
The validation of the derived critical coupling strengths. The derived results are compared to the simulation results. The prediction of the derived results get more accurate if the points are closer to the diagonal line $y=x$. There are three groups of network sizes, which are indicated by signs: (1) ‘$\circ$’, ${N}^{I}=80$, ${N}^{II}=100$; (2) ‘+’, ${N}^{I}=120$, ${N}^{II}=100$; (3) ‘$\diamond$’, ${N}^{I}=150$, ${N}^{II}=100$. Seven colors present the results of seven connection strategies respectively. For example, blue for **H**-**L L**-**L**. (**a**) The validation of derived *λ*_*c*_ for seven connection strategies. (**b**) The validation of derived *β*_*c*_ for seven connection strategies. (**c**) For all three groups of network sizes, we choose three values of *λ*, which are indicated by the size of sign and bigger value yields larger size. Group 1: 1.13; 1.14; 1.15. Group 2: 0.99; 1.00; 1.01. Group 3: 0.985; 0.987; 0.989.

The derived critical coupling strengths are validated in Fig. 5. The x-label in Fig. 5 describes the results obtained by our theory (using superscript **T**), while the y-label denotes the simulation results (using superscript **S**). When the derived results are as the same as the simulation results, the corresponding dots are on the diagonal line $y=x$. Three kinds of signs indicate three groups of network sizes: (1) ‘$\circ$’, ${N}^{I}=80$, ${N}^{II}=100$; (2) ‘+’, ${N}^{I}=120$, ${N}^{II}=100$; (3) ‘$\diamond$’, ${N}^{I}=150$, ${N}^{II}=100$, while seven colors represent the results of seven connection strategies, such as blue for **H**-**L L**-**L** etc. In Fig. 5a–c we compare results from our theory with ones from the simulations for *λ*_*c*_, *β*_*c*_ and *β*_*max*_ respectively. The comparisons show that our theory aligns with the simulation.

### Phase diagram

The above analysis points out the significant role of network sizes for the synchronization of NONs. In real systems, the network sizes are not constant and may change over time. Thus, it’s important to develop an approach to keep the system synchronized in a fast and efficient way, facing the time-variable network sizes.

First of all, we introduce a concept of “maximum allowable network sizes”, which comes from “maximum allowable penetration level”, a hot spot in the field of smart grid^45^. The “maximum allowable network sizes” refers to the maximum network sizes that the system can tolerate. If the network sizes are larger than this value, the system fails to remain synchronized. For **H**-**H**, the maximum allowable network sizes can be obtained from equation (3). Assuming ${N}^{I}\ge {N}^{II}$ without loss of generation, we have

12 $${N}^{II}\le -\,{N}^{I}+\frac{4\omega }{\omega -\lambda },\lambda  < \omega ,$$

13 $${N}^{II}\ge \frac{2\omega -\beta }{2\omega +\beta }{N}^{I}.$$

It is noticed that when $\lambda \geqslant \omega$, the maximum allowable network size of Network II is $\frac{2\omega -\beta }{2\omega +\beta }{N}^{I}$, only determined by *β*. Similarly, the maximum allowable network sizes for other cases can also be obtained.

**Figure 6:**
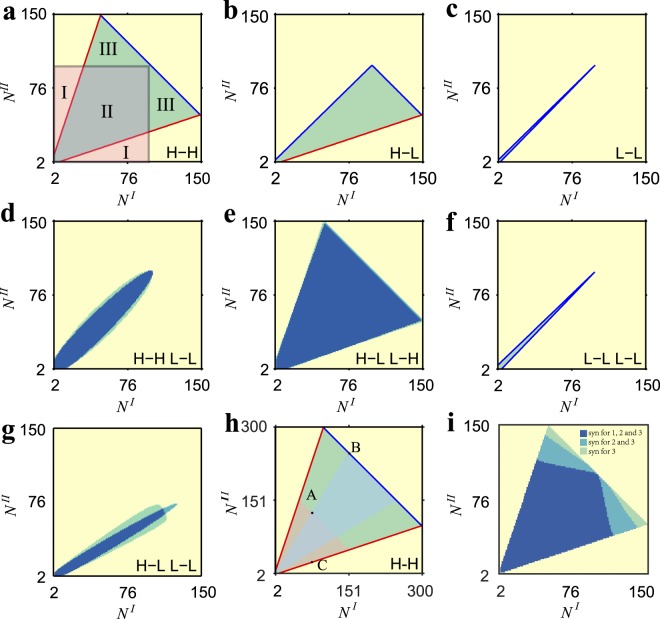
The operating space constructed with network sizes. (**a**) The synchronization area of two uncoupled networks is shown as the translucent red zone (zone I and zone II), while that of two networks coupled through **H**-**H** is shown as green zone (zone II and zone III), with $\lambda =0.98$, $\beta =1.00$. In (**d**, **e** and **g**),  indicates the system with *N*^*I*^ and *N*^*II*^ is proved to be non-synchronized by both the theory and the simulation,  indicates this system is proved to be synchronized by both the theory and the simulation,  indicates this system is proved to be synchronized by the simulation but not by the theory, and  indicates this system is proved to be synchronized by the theory but not by the simulation. (**h**) Green zone presents the operating space for $\lambda =0.99$, $\beta =1.00$, red zone denotes that for $\lambda =0.98$, $\beta =1.00$, and blue zone shows that for $\lambda =0.99$, $\beta =1.00$. $A(73,122)$, $B(245,150)$ and $C(73,23)$. (**i**) Three groups of synchronization area: (1) *S*_1_ for the uniform networks with $\lambda =0.98$; (2) *S*_2_ for the non-uniform networks with *λ* randomly chosen from $(0.98,0.99)$; (3) *S*_3_ for the uniform networks with *λ* continuously drops from 1.27 to 0.98 (${S}_{3}\mathop{\supset }\limits_{\ne }{S}_{2}\mathop{\supset }\limits_{\ne }{S}_{1}$).  represent ${S}_{1}\cup {S}_{2}\cup {S}_{3}$,  stands for ${S}_{2}\cup {S}_{3}$, while  shows *S*_3_.

With certain coupling strengths, whether the system with *N*^*I*^ and *N*^*II*^ remains synchronized or not is shown by the phase diagram. As seen in Fig. 6a–g, the phase diagram includes two part: one is the operating space (green or blue zone), constructed by the allowable network sizes; the other is the non-synchronized area (yellow zone), constructed by the non-allowable network sizes. The results, again, show that our theoretical results (blue and red lines in Fig. 6a–c and f, green zone in Fig. 6d, e and g) align with simulation results (green zone in Fig. 6a–c and f, blue zone in Fig. 6d, e and g).

### Network Sizes

Comparing the operating spaces from different connection strategies, we find that the optimal connection strategy depends on the network sizes, contradicting to the previous findings^25,26^ that connecting the high-degree nodes is always the most competitive one. As seen in Fig. 6f and g, when ${N}^{I}\approx {N}^{II}$, the system with **L**-**L L**-**L** (green zone) is much easier to be synchronized than that with **H**-**L L**-**L** (yellow zone), although the system with **H**-**L L**-**L** has a high-degree node as the connector. Besides, all the connection strategies except **H**-**L L**-**L** share similar operating space when ${N}^{I}\approx {N}^{II}$, *i*.*e*. near the diagonal line in Fig. 6a–g. One more example is that, **H**-**H L**-**L** connects the high-degree nodes while **H**-**L L**-**H** connects the high-degree node to the low-degree node. However, the former (Fig. 6d) has much less operating space than the later one (Fig. 6e). Counter-intuitively, more links between networks do not definitely lead to more operating space. For example, the operating space of **H**-**H** is much larger that that of **H**-**H L**-**L**, although the later has one more link.

It’s natural to ask further: how does the interdependence influences the operating space? Will interconnected networks be more stable than a single network? As shown in Fig. 6a, the operating space^40^ of a single network is zone I and zone II, while that for networks coupled by hubs is zone II and zone III. If Network I and Network II belong to zone I, they will lose synchronization if they become interdependent on each other. If Network I and Network II belong to zone III, they will lose synchronization if they loss interdependence. So whether the interdependence enhances the synchronization or not depends on the zone that the system belong to, *i*.*e*., the combination of the network sizes. This finding can inform whether it would be helpful to integrate different networks or not. The integration should be stopped if the interdependence brought by it drives the system from the operating space into the unsynchronized zone.

Facing the fast-changed network sizes, our results contribute to the time-demanding control. With certain coupling strengths ($\lambda =0.98$, $\beta =0.50$), the system $A(73,122)$ is synchronized, as seen in Fig. 6h (*A* belongs to the intersection of red zone and blue zone: red zone, the safe operating space for $\lambda =0.98$, $\beta =1.00$; blue zone, the safe operating space for $\lambda =0.98$, $\beta =1.00$). With the enlargement of network sizes, $A(73,122)$ jumps into $B(245,150)$, the system cannot maintain synchronized (green zone, the safe operating space for $\lambda =0.99$, $\beta =1.00$). To maintain synchronization, it is *λ* that should be enhanced, while the enhancement of *β* does not make sense. Surprisingly, when the network size is decreased, $A(73,122)$ jumps to $C(73,23)$, we need to increase instead decrease *β*. These results highlight the distinct roles of the internal coupling strength (*λ*) and the external coupling strength (*β*) on operating space; the former one sizes the operating space while the latter, shapes. This finding helps to decide which coupling strength should be utilized and how much it should be enhanced, guiding the time-demanding control to maintain synchronization.

**Figure 7:**
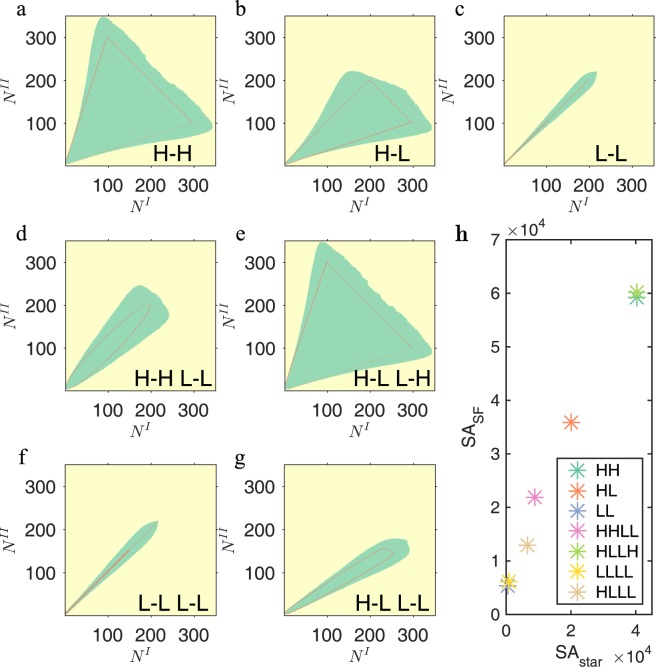
The validation on SF networks. The result derived by star networks is validated in SF networks through the comparison of phase diagram with the same coupling strength $\lambda =0.99$, $\beta =1.00$. SF network is grown by the preferential attachment with one link for a new node and two networks share the same growing process. In all plots, the synchronization area of star network is the one surrounded by red curves, while the synchronization area of SF networks is the green zone. The seven subplots are for seven connection strategies respectively: (**a**) **H**-**H**. (**b**) **H**-**L**. (**c**) **L**-**L**. (**d**) **H**-**H L**-**L**. (**e**) **H**-**L L**-**H**. (**f**) **L**-**L L**-**L**. (**g**) **H**-**L L**-**L**. (**h**) We calculate the acreage of the operating space of both star networks and SF networks, for all connection strategies.

The theoretical results are verified on the heterogeneous networks. In Fig. 6i, there are three groups of phase diagrams with $\beta =1.00$: Group 1 indicates the operating space with $\lambda =0.98$, at which the synchronized systems loss synchronization; Group 2 concerns the unsynchronized systems that will gain synchronization with $\lambda =0.98$; Group 3 is a sensitivity test (see Method Sensitivity Tests) with *λ* randomly chosen from $[0.98,0.99]$. It can be seen that the synchronization area of Group 3 (unsynchronized to synchronized, $\lambda \in [0.98,0.99]$) is very close to that of Group 1 (synchronized to unsynchronized, $\lambda =0.99$). Moreover, our theoretical predictions of the synchronized regimes agrees with the simulation results on SF networks, as shown in Fig. 7. In conclusion, our result derived through homogenous networks can be applied to the heterogeneous ones.

## Discussion

In this paper, we introduce a mathematical method to study the synchronization without lossing inherent nonlinearity. Since the method avoids the usage of super-Laplacian matrix, it can handle NONs with massive layers, especially for time-demanding control.

The method enabled us to, for the first time, discover the importance of network sizes in the study of the synchronization of NONs. Firstly, it is found that the optimal connection strategy depends on the network sizes. As we show in Fig. 6d and e, the operating space of **H**-**H L**-**L** is much smaller than that of **H**-**L L**-**H**. The finding, contradicting to the previous opinion that to connect the nodes with high degree is always the optimal solution, provides new insights to develop optimal connecting strategies. Secondly, the network sizes shed new light on the debate that whether the interdependence affects the stability negatively or positively. We found interdependence plays a positive role when the sizes between the networks are similar, and a negative role when the sizes are different, as seen in Fig. 6a. Thirdly, we find that network sizes help to guide the fast control to maintain synchronization. Network sizes indicates the kind of coupling strength that should be adjusted and the amount of adjustments that should be made, as seen in Fig. 6h.

The importance of network sizes to the synchronization of NONs will have important practical applications. The high penetration level of new energy source, *i*.*e*. grid networks of extremely varying sizes, should be carefully monitored. The decision-making of integrating microgrids into the main power grid should depend on whether the interdependence drives the system out of synchronization. The adjustments of coupling strengths need to follow the change in network sizes over time to keep the synchronization of smart-grids with time-varying penetration level. It is worth noticing that the applications we gave here is through the modeling of smart-grid as oscillator-based^18^ NONs. Controlling real smart grids with more complex network structures is more challenging and requires further research.

Despite the diverse properties and complex behaviors of NONs, we find a universal characteristic that dominates the synchronization of NONs: “***unbalance***”. If there is an unbalance between the network sizes, *i*.*e*., ${N}_{1}\gg {N}_{2}$, the synchronization level is much lower than a “***balanced***” system with ${N}_{1}={N}_{2}$ for a given *β*. Also, intuitively we would assume that connection strategy of **H**-**H L**-**L** should result a larger synchronized space than **H**-**H** due to the extra linkage. However, the result is opposite, as seen in Fig. 6a and d. This is because the additional link **L**-**L** makes the networks more “***unbalanced***”. Take **H**-**H** in Fig. 7a for example. When the difference between network sizes gets larger, the unbalance between the two star networks’ structures gets larger, while the two SF networks get more balanced due to the randomness. Thus, we may draw the conclusion that the unbalance between networks is a universal factor obstructing the synchronization of interconnected networks. Surprisingly, when we calculate the sizes of the operating space between star networks (SA_*star*_) and SF networks (SA_*SF*_), the ratios of different connection strategies are almost along a universal line (Fig. 7h). Assuming the existence of this ‘universal’ line, we can obtain the information of SF networks without calculations once we know the results of the corresponding star networks. That is to say, despite the fact that the topology of the SF networks is much different from that of the star networks, they share some universal properties. However, the existence of the potential universality needs further exploration.

## Methods

### Kuramoto model

The mathematical description of the Kuramoto model^37^ is as

14 $${\dot{\theta }}_{i}={\omega }_{i}-\sum _{j}\,{\lambda }_{ij}\,\sin \,({\theta }_{i}-{\theta }_{j}).$$

Each node characterized by a phase angle *θ*_*i*_ has the intrinsic rotation frequency ${\omega }_{i}$, but is limited by the coupled neighbor through an elastic spring with stiffness *λ*_*ij*_.

### Coupled Kuramoto model

The coupled Kuramoto model for Network I and Network II is

15 $$\begin{array}{rcl}{\dot{\theta }}_{i}^{I} & = & {\omega }_{i}^{I}-\sum \,{\lambda }_{ij}^{I}\,\sin \,({\theta }_{i}^{I}-{\theta }_{j}^{I})-\sum \,{\beta }_{ip}^{I}\,\sin \,({\theta }_{i}^{I}-{\theta }_{p}^{II}),\\ {\dot{\theta }}_{m}^{II} & = & {\omega }_{m}^{II}-\sum \,{\lambda }_{mn}^{II}\,\sin \,({\theta }_{m}^{II}-{\theta }_{n}^{II})-\sum \,{\beta }_{mk}^{II}\,\sin \,({\theta }_{m}^{II}-{\theta }_{k}^{I}),\end{array}$$

where $\theta \in [0,2\pi )$ is the phase of a node, and the natural frequency is proportional to its degree^41^, *i*.*e*., ${\omega }_{i}^{I}={k}_{i}^{I}\omega$, ${\omega }_{m}^{II}={k}_{m}^{II}\omega$. Here $\omega$ is the natural rotating frequency unit and *k* is the degree of the node. Different from single network, there are two kinds of coupling strengths for interconnected networks. One is the coupling strength within a network (the internal coupling strength), *λ*. The other is the coupling strength between networks (the external coupling strength), *β*.

### Parameter setup

The coupled Kuramoto model was applied to the simulations shown in Figs 2, 5, 6 and 7, with the initial condition of the phases of all oscillators as 1. Then *λ* drops gradually and everytime *λ* changes, the initial phases are the end states of the last *λ*. In all these plots, the natural frequency of all oscillators were 1 and *β* remained unchanged. *λ* was a fixed value in the simulation shown in Figs 2a–e and 5, while in Figs 2f–i, 6 and 7 *λ* was kept unchanged for 10000 iterations and passed to another value for another 100000 iterations with $\delta \lambda =-\,0.01$. Fourth-order Runge-Kutta was used in the iteration to solve the nonlinear equations.

### Scale-Free Networks

Scale-Free network was generated using BB model^46^. The initial network had one node. A new node was added to a existed node *i* with the probability ${{\rm{\Pi }}}_{i}$, where ${{\rm{\Pi }}}_{i}=\frac{{\eta }_{i}{k}_{i}}{{\sum }_{j}\,{\eta }_{j}{k}_{j}}$. Here *k* is the degree of one node and the mean degree is 1. *η* is the fitness randomly chosen between $[0,1]$ for every node.

### Connection Strategy and Adjacency Matrix

Connection strategy refers to which node acts as the connector that linking different networks. All the connections between two networks discussed in this study are **H**-**H** (short for hub-hub, means two hubs from two networks respectively act as such connectors,), **H**-**L** (short for hub-leaf), **L**-**L**, **H**-**H L**-**L**, **H**-**L L**-**H**, **L**-**L L**-**L**, and **H**-**L L**-**L**. Note that our results can be extended to multiple couplings between two networks. Connection Strategy indicates the adjacency matrix between networks *M*. When the nodes *i* and *j* act as the connector linking two networks, then ${M}_{ij}=1$, else ${M}_{ij}=0$.

### Sensitivity Tests

The derived results were validated on the heterogeneous networks, as shown in Fig. 7i. We used three groups of coupling strengths: (1) all nodes were assigned with $\lambda =0.98$; (2) for each node, *λ* were randomly chosen from $[0.98,0.99]$; (3) *λ* for all nodes started from 1.20 that the system could be synchronized, then *λ* for all nodes deceased gradually until 0.98 to check whether the system could be synchronized or not.

## Supplementary information

Supplementary Information

## Data Availability

The datasets generated and/or analysed in this study are available from the corresponding author on reasonable request.
