# Supplementary material for: Synchronization of interconnected heterogeneous networks: The role of network sizes
Source: Sci Rep. 2019 Apr 16;9:6154. doi: 10.1038/s41598-019-42636-6 (PMC6468008; doi:10.1038/s41598-019-42636-6)
Supplement: Supplementary file 1 — Supplementary Information [file 41598_2019_42636_MOESM1_ESM.pdf]

Supplementary Information

Synchronization of interconnected heterogeneous networks: The role of network sizes
by Huixin Zhang, Weidong Zhang, Jianxi Gao

Table of Contents

S1 Dynamics in the integrated grid **2**

S2 One-link connection **2**
S2.1 Case H-H . . . 3
S2.2 Case H-L . . . 4
S2.3 Case L-L . . . 5

S3 Two-links connection **8**
S3.1 Case H-H L-L . . . 9
S3.2 Case H-L L-H . . . 13
S3.3 Case L-L L-L . . . 17
S3.4 Case H-L L-L . . . 19

S4 A special case: the two network sizes are equal **30**

S5 Different roles between internal and external coupling strengths **31**

S6 Phase diagram composed of hub degrees **33**

List of Figures

S1 Critical coupling strengths in **H-H** case . . . 7
S2 Critical coupling strengths in **H-L** case . . . 7
S3 Critical coupling strengths in **L-L** case . . . 8
S4 The critical internal coupling strength in two-links connection . . . 28
S5 The critical external coupling strength in two-links connection . . . 29
S6 Phase diagram with $\lambda = 0.99, \beta = 1.00$ . . . 32
S7 Phase diagram with $\lambda = 0.99, \beta = 0.50$ . . . 32
S8 Phase diagram based on combinations of K^I and K^{II} . . . 33

List of Tables

S1 The endpoints in **H-H L-L** . . . 10
S2 The endpoints in **H-L L-H** . . . 14
S3 The endpoints in **H-L L-L** . . . 21
S4 The equal network sizes. . . . 30

S1 Dynamics in the integrated grid

We consider an integrated grid composed of a traditional power grid and a microgrid, with N^I and N^{II} nodes respectively. The dynamics governing the integrated grid can be written as

$$\begin{aligned} \dot{\theta}_i^I &= \omega_i^I - \sum \lambda_{ij}^I \sin\left(\theta_i^I - \theta_j^I\right) - \sum \beta_{ip}^I \sin\left(\theta_i^I - \theta_p^{II}\right), \\ \dot{\theta}_m^{II} &= \omega_m^{II} - \sum \lambda_{mn}^{II} \sin\left(\theta_m^{II} - \theta_n^{II}\right) - \sum \beta_{mk}^{II} \sin\left(\theta_m^{II} - \theta_k^I\right), \end{aligned} \tag{S1}$$

where $\theta \in [0, 2\pi)$ denotes the phase of a node as a time dependent activity, I (II) means the node belongs to Network I (Network II) with $i = 1, ..., N^I$ ($m = 1, ..., N^{II}$), ω stands for the natural frequency, λ captures the interaction between nodes that have one link in the same network as internal coupling strength, and β encapsulates the interaction between nodes connecting the two networks as external coupling strength. The grid frequency ω is a fixed value, $50Hz$ or $60Hz$, and ω is proportional to its degree k[1], i.e. $\omega_i^I = k_i^I\omega$, $\omega_m^{II} = k_m^{II}\omega$. For internal coupling strength, $\lambda_{ij}^I = V_i^I V_j^I \Im\left(Q_{red}[i, j]\right) = \lambda_{ji}^I$ in the traditional power grid[2] and $\lambda_{mn}^{II} = V_m^{II} V_n^{II} |Y_{mn}| = \lambda_{nm}^{II}$ in the microgrid with the frequency-droop controller[3], where V is the voltage, Y accounts for the admittance and Q_{red} refers to the reduced admittance matrix. As for external coupling strength, $\beta_{ip}^I = V_i^I V_p^{II} \left|Y_{ip}^{I,II}\right| = V_p^{II} V_i^I \left|Y_{pi}^{II,I}\right| = \beta_{pi}^{II}$ ($\beta_{mk}^{II} = V_m^{II} V_k^I \left|Y_{mk}^{II,I}\right| = V_k^I V_m^{II} \left|Y_{mk}^{I,II}\right| = \beta_{km}^I$) in the integrated grid. Here, we study the uniform networks with the dynamical equations

$$\begin{aligned} \dot{\theta}_i^I &= k_i^I\omega - \lambda \sum \sin\left(\theta_i^I - \theta_j^I\right) - \beta \sum \sin\left(\theta_i^I - \theta_p^{II}\right), \\ \dot{\theta}_m^{II} &= k_m^{II}\omega - \lambda \sum \sin\left(\theta_m^{II} - \theta_n^{II}\right) - \beta \sum \sin\left(\theta_m^{II} - \theta_k^I\right). \end{aligned} \tag{S2}$$

The application of the result derived by the uniform networks (S2) can be well applied to the non-uniform networks (S1), seen in the manuscript Fig. 4**i**.

S2 One-link connection

Before the construction of a microgrid, the experts need to determine how the microgrid will be integrated into the traditional power grid. We suggest that the topology between the microgrid and the traditional power grid is the factor that needs to be considered. The topology between them is called the connection strategy, which is found to be very important according to the previous research[4,5]. The connection strategy for two networks coupled through one link has three cases: **H-H**, **H-L** and **L-L**[4].

S2.1 Case H-H

When the system is synchronized, the two networks are in the state of locking manifolds[6]. The locking manifolds in **H-H** case can be expressed as

$$\begin{aligned} \mathrm{M}_1 &= \left\{\theta^I_{N^I} - \theta^I_i = a, i = 1, ..., N^I - 1, a \in [0, 2\pi)\right\}, \\ \mathrm{M}_2 &= \left\{\theta^{II}_{N^{II}} - \theta^{II}_m = b, m = 1, ..., N^{II} - 1, b \in [0, 2\pi)\right\}, \end{aligned} \tag{S3}$$

where a and b are the constants relating with the phase difference between the hub and the leaf. Since all leaves in the star motif are exactly the same, so a and b are the scalar[6].Here the node N^I is the hub in Network I while the node N^{II} is the hub in Network II, same for the other cases. Using locking manifolds, (S2) for the synchronized system here can be rewritten as

$$\begin{aligned} \dot\theta^I_i &= \omega + \lambda \sin a, i = 1, ..., N^I - 1, \\ \dot\theta^I_{N^I} &= \left(N^I - 1\right)\omega - \lambda\left(N^I - 1\right)\sin a - \beta \sin\left(\theta^I_{N^I} - \theta^{II}_{N^{II}}\right), \\ \dot\theta^{II}_m &= \omega + \lambda \sin b, m = 1, ..., N^{II} - 1, \\ \dot\theta^{II}_{N^{II}} &= \left(N^{II} - 1\right)\omega - \lambda\left(N^{II} - 1\right)\sin b - \beta \sin\left(\theta^{II}_{N^{II}} - \theta^I_{N^I}\right). \end{aligned} \tag{S4}$$

Since the phase difference between $\theta_{\mathtt{hub}}$ and $\theta_{\mathtt{leaf}}$ does not change with time, we have $\dot\theta^I_{N^I} - \dot\theta^I_i = 0$ and $\dot\theta^{II}_{N^{II}} - \dot\theta^{II}_m = 0$. Substituting $\dot\theta^I_{N^I} - \dot\theta^I_i = 0$ and $\dot\theta^{II}_{N^{II}} - \dot\theta^{II}_m = 0$ into (S4), we get

$$\begin{aligned} &\lambda = \frac{N^I + N^{II} - 4}{N^I \sin a + N^{II} \sin b}\omega, \\ &\left(N^I - 2\right)\omega - \lambda N^I \sin a - \beta \sin\left(\theta^I_{N^I} - \theta^{II}_{N^{II}}\right) = 0, \\ &\left(N^{II} - 2\right)\omega - \lambda N^{II} \sin b - \beta \sin\left(\theta^{II}_{N^{II}} - \theta^I_{N^I}\right) = 0. \end{aligned} \tag{S5}$$

Since $\theta^I_{N^I} - \theta^{II}_{N^{II}}$ is a constant, $\theta^I_{N^I} - \theta^I_j$ and $\theta^I_{N^{II}} - \theta^{II}_j$ are constants too. Thus, (S4) yields

$$\sin a = \sin b = \frac{N^I + N^{II} - 4}{\lambda\left(N^I + N^{II}\right)}\omega, \tag{S6}$$

$$\sin\left(\theta^I_{N^I} - \theta^{II}_{N^{II}}\right) = \frac{2\left(N^I - N^{II}\right)}{\beta\left(N^I + N^{II}\right)}\omega. \tag{S7}$$

As $\sin a$ and $\sin\left(\theta^I_{N^I} - \theta^{II}_{N^{II}}\right)$ in the l.h.s of (S6) and (S7) are limited in $[-1, 1]$, the r.h.s of (S6) and (S7) are limited in $[-1, 1]$ too. Hence, the range of λ and β can be obtained as

$$\lambda \geqslant \frac{N^I + N^{II} - 4}{N^I + N^{II}}\omega, \tag{S8}$$

$$\beta \geqslant \frac{2\left|N^I - N^{II}\right|}{N^I + N^{II}}\omega, \tag{S9}$$

namely,

$$\lambda_c^{\textbf{H-H}} = \frac{N^I + N^{II} - 4}{N^I + N^{II}}\omega, \tag{S10}$$

$$\beta_c^{\textbf{H-H}} = \frac{2\left|N^I - N^{II}\right|}{N^I + N^{II}}\omega. \tag{S11}$$

S2.2 Case H-L

When the two networks are coupled through **H-L** connection, there exits three locking manifolds for the synchronized system:

$$\begin{aligned}
\mathrm{M}_1 &= \left\{\theta^I_{N^I} - \theta^I_i = a, i = 1, ..., N^I - 1, a \in [0, 2\pi)\right\}, \\
\mathrm{M}_2 &= \left\{\theta^{II}_{N^{II}} - \theta^{II}_m = b, m = 2, ..., N^{II} - 1, b \in [0, 2\pi)\right\}, \\
\mathrm{M}_3 &= \left\{\theta^{II}_{N^I} - \theta^{II}_1 = d, d \in [0, 2\pi)\right\},
\end{aligned} \tag{S12}$$

where the connectors coupling two networks are the hub node N^I and the leaf node 1^{II}, a, b and c are the constants relating with the phase difference between the hub and the leaf. With the locking manifolds (S12), the dynamics of the synchronized system is

$$\begin{aligned}
\dot{\theta}^I_i &= \omega + \lambda \sin a, i = 1, ..., N^I - 1 \\
\dot{\theta}^I_{N^I} &= \left(N^I - 1\right)\omega - \lambda\left(N^I - 1\right)\sin a - \beta \sin\left(\theta^I_{N^I} - \theta^{II}_1\right), \\
\dot{\theta}^{II}_1 &= \omega + \lambda \sin d - \beta \sin\left(\theta^{II}_1 - \theta^I_{N^I}\right), \\
\dot{\theta}^{II}_m &= \omega + \lambda \sin b, m = 2, ..., N^{II} - 1 \\
\dot{\theta}^{II}_{N^{II}} &= \left(N^{II} - 1\right)\omega - \lambda\left(N^{II} - 2\right)\sin b - \lambda \sin d.
\end{aligned} \tag{S13}$$

According to the locking manifolds (S12), $\dot{\theta}^I_{N^I} - \dot{\theta}^I_i = 0$, $\dot{\theta}^{II}_{N^{II}} - \dot{\theta}^{II}_m = 0$ and $\dot{\theta}^{II}_{N^I} - \dot{\theta}^{II}_1 = 0$. Thus, from (S13) we have $\dot{\theta}^I_i = \dot{\theta}^I_{N^I} = \dot{\theta}^{II}_1 = \dot{\theta}^{II}_m = \dot{\theta}^{II}_{N^{II}}$ and

$$\sin a = \sin b = \frac{N^I + N^{II} - 4}{\lambda\,(N^I + N^{II})}\omega, \tag{S14}$$

$$\sin d = \frac{-N^I + 3N^{II} - 4}{\lambda\,(N^I + N^{II})}\omega, \tag{S15}$$

$$\sin\left(\theta^I_{N^I} - \theta^{II}_1\right) = \frac{2\left(N^I - N^{II}\right)}{\beta\,(N^I + N^{II})}\omega. \tag{S16}$$

The ranges of the sine values in (S14-S16) determine the ranges of the coupling strengths

$$\lambda \geqslant \frac{N^I + N^{II} - 4}{N^I + N^{II}}\omega, \tag{S17}$$

$$\lambda \geqslant \frac{-N^I + 3N^{II} - 4}{N^I + N^{II}}\omega, \tag{S18}$$

$$\beta \geqslant \frac{2\left(N^I - N^{II}\right)}{N^I + N^{II}}\omega, \tag{S19}$$

leading to

$$\lambda_c^{\textbf{H-L}} = \begin{cases} \dfrac{N^I + N^{II} - 4}{N^I + N^{II}}\omega, & N^I \geqslant N^{II} \\ \dfrac{-N^I + 3N^{II} - 4}{N^I + N^{II}}\omega, & N^I < N^{II} \end{cases}, \tag{S20}$$

$$\beta_c^{\textbf{H-L}} = \frac{2\left|N^I - N^{II}\right|}{N^I + N^{II}}\omega. \tag{S21}$$

S2.3 Case L-L

The locking manifolds for the synchronized system in **L-L** case can be expressed as

$$\begin{aligned} \mathrm{M}_1 &= \left\{\theta^I_{N^I} - \theta^I_i = a, i = 2, ..., N^I - 1, a \in [0, 2\pi)\right\}, \\ \mathrm{M}_2 &= \left\{\theta^{II}_{N^{II}} - \theta^{II}_m = b, m = 2, ..., N^{II} - 1, b\, [0, 2\pi)\right\}, \\ \mathrm{M}_3 &= \left\{\theta^{II}_{N^I} - \theta^I_1 = c, c \in [0, 2\pi)\right\}, \\ \mathrm{M}_4 &= \left\{\theta^{II}_{N^{II}} - \theta^{II}_1 = d, d \in [0, 2\pi)\right\}, \end{aligned} \tag{S22}$$

where the leaf node 1^I and the leaf node 1^{II} are the connectors linking two networks, a, b, c and d are the constant phase differences. Substituting the locking manifolds (S22) into (S2) for **L-L** case, we have

$$\sin a = \sin b = \frac{N^I + N^{II} - 4}{\lambda\,(N^I + N^{II})}\omega, \tag{S23}$$

$$\sin c = \frac{-N^I + 3N^{II} - 4}{\lambda\,(N^I + N^{II})}\omega, \tag{S24}$$

$$\sin d = \frac{3N^I - N^{II} - 4}{\lambda\,(N^I + N^{II})}\omega, \tag{S25}$$

$$\sin\left(\theta^I_1 - \theta^{II}_1\right) = \frac{2\left(N^I - N^{II}\right)}{\beta\,(N^I + N^{II})}\omega. \tag{S26}$$

Thus, the critical coupling strengths are

$$\lambda_c^{\textbf{L-L}} = \begin{cases} \dfrac{3N^I - N^{II} - 4}{N^I + N^{II}}\omega, & N^I \geqslant N^{II} \\ \dfrac{-N^I + 3N^{II} - 4}{N^I + N^{II}}\omega, & N^I < N^{II} \end{cases}, \tag{S27}$$

$$\beta_c^{\textbf{L-L}} = \frac{2\left|N^I - N^{II}\right|}{N^I + N^{II}}\omega. \tag{S28}$$

Through the comparison of λ_c in the three cases above, we find that $\lambda_c^{\textbf{H-H}} \geqslant \lambda_c^{\textbf{H-L}} \geqslant \lambda_c^{\textbf{L-L}}$. More hub acts as the connector, less the critical value of internal coupling strength. Moreover, it is surprised to notice that the critical values are equal when the two network sizes are the same, i.e. $\lambda_c^{\textbf{H-H}} = \lambda_c^{\textbf{H-L}} = \lambda_c^{\textbf{L-L}}$ when $N^I = N^{II}$, which is verified in S4. As for external coupling strength, β_c for all the modes in one-link connection are the same. That means whether hub acts as the connector or not doesn't influence external coupling strength. In sum, hub plays a significant role in internal coupling strength, but has no effect on external coupling strength.

We ran (S2) with $N^I = 11, N^{II} = 16, \omega = 1$ for case **H-H** (Fig. S1), case **H-L** (Fig. S2) and case **L-L** (Fig. S3) respectively. In each figure, there are four sub-figures with the same conditions except the coupling strengths: **a**, $\lambda_c - 0.01, \beta_c - 0.01$; **b**, $\lambda_c - 0.01, \beta_c + 0.01$; **c**, $\lambda_c + 0.01, \beta_c - 0.01$; **d**, $\lambda_c + 0.01, \beta_c + 0.01$. Only the phase differences with $\lambda_c + 0.01$ and $\beta_c + 0.01$ shown in **d** can remain unchanged as time goes. The critical points "λ_c" and "β_c" mentioned above are obtained through the simulations, which separates the synchronized and non-synchronized phase. The exact values of λ_c are 0.87 (Fig. S1), 0.87 (Fig. S2) and 1.23 (Fig. S3), which are very close to the derived results 0.86 obtained from (S10), 0.86 obtained from (S20) and 1.23 obtained from (S27) respectively. And the exact values of β_c are 0.37 in Figs. S1-S3, the same as the derived results from (S11), (S21) and (S28). These very close values verify the result derived in S2.

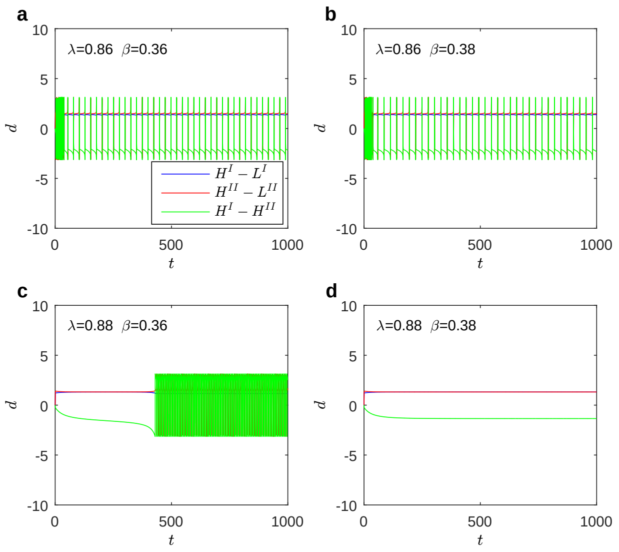

Figure S1: Critical coupling strengths in **H-H** case. We ran Kuramoto dynamic (S2) on two networks coupled through **H-H** connection with $N^I = 11, N^{II} = 16, \omega = 1$. Phase differences include the one between the hub and the leaf (blue curves for Network I and red curves for Network II) and the one between two hubs (green curves). **a**, With $\lambda = 0.86, \beta = 0.36$, phase differences change with time. Similar results are found in **b** ($\lambda = 0.86, \beta = 0.38$) and **c** ($\lambda = 0.88, \beta = 0.36$). **d**, Phase differences are invariant with $\lambda = 0.88, \beta = 0.38$.

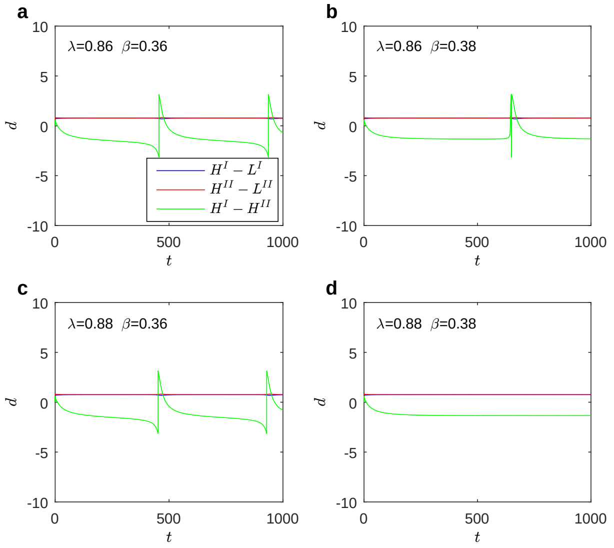

Figure S2: Critical coupling strengths in **H-L** case. The Kuramoto dynamic (S2) was cast on two networks coupled through **H-L** connection with $N^I = 11, N^{II} = 16, \omega = 1$. Phase differences include the one between the hub and the leaf (not the leaf connector, blue curves for Network I and red curves for Network II) and the one between two hubs (green curves). **a**, With $\lambda = 0.86$, $\beta = 0.36$, phase differences change with time. Similar results are found in **b** ($\lambda = 0.86, \beta = 0.38$) and **c** ($\lambda = 0.88, \beta = 0.36$). **d**, Phase differences are invariant with $\lambda = 0.88, \beta = 0.38$.

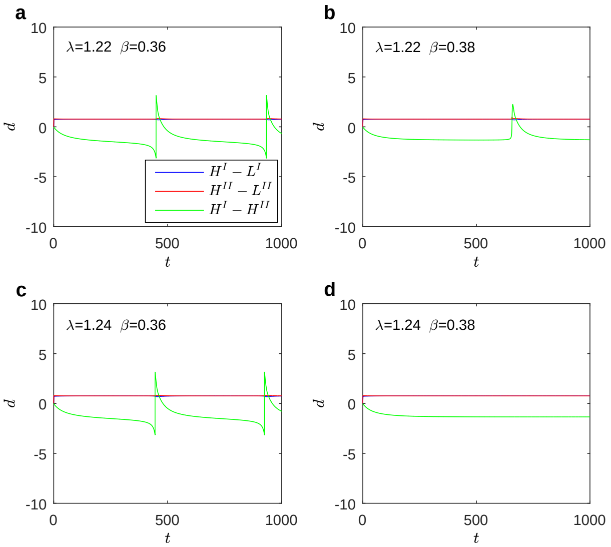

Figure S3: Critical coupling strengths in **L-L** case. Using (S2) on two networks in case **L-L** with $N^I = 11, N^{II} = 16, \omega = 1$, we get how the phase differences change with time. Phase differences include the one between the hub and the leaf (not the leaf connector, blue curves for Network I and red curves for Network II) and the one between two hubs (green curves). **a**, With $\lambda = 1.22$, $\beta = 0.36$, the phase differences change with time. Similar results are found in **b** ($\lambda = 1.22$, $\beta = 0.38$) and **c** ($\lambda = 1.24, \beta = 0.36$). **d**, Phase differences are invariant with $\lambda = 1.24, \beta = 0.38$.

S3 Two-links connection

In two-links connection, the connection strategies investigated for the integrated grid are **H-H L-L**, **H-L L-H**, **L-L L-L** and **H-L L-L**. To get the expression of the critical coupling strengths here, Taylor approximation is used. The use of it is based on the fact that both the phases of the converter and the generator need to be very close to the phase of the injection points, suggesting that all the phase differences are close to zero[7].

S3.1 Case H-H L-L

The locking manifolds in case **H-H L-L** are as same as (S22) in case **L-L**. Using the locking manifolds we can write (S2) for the synchronized system as

$$
\begin{aligned}
\dot{\theta}_1^I &= \omega + \lambda \sin c - \beta \sin\left(\theta_1^I - \theta_1^{II}\right), \\
\dot{\theta}_i^I &= \omega + \lambda \sin a, i = 2, .., N^I - 1, \\
\dot{\theta}_{N^I}^I &= \left(N^I - 1\right)\omega - \lambda\left(N^I - 2\right)\sin a - \lambda \sin c - \beta \sin\left(\theta_{N^I}^I - \theta_{N^{II}}^{II}\right), \\
\dot{\theta}_1^{II} &= \omega + \lambda \sin d - \beta \sin\left(\theta_1^{II} - \theta_1^I\right), \\
\dot{\theta}_m^{II} &= \omega + \lambda \sin b, m = 2, .., N^{II} - 1, \\
\dot{\theta}_{N^{II}}^{II} &= \left(N^{II} - 1\right)\omega - \lambda\left(N^{II} - 2\right)\sin b - \lambda \sin d - \beta \sin\left(\theta_{N^{II}}^{II} - \theta_{N^I}^I\right),
\end{aligned}
\tag{S29}
$$

where the hub node N^I connects to the hub node N^{II} and the leaf node 1^I connects to the leaf node 1^{II}. Since the phase differences are constants, $\dot{\theta}_{N^I}^I - \dot{\theta}_1^I = 0$, $\dot{\theta}_{N^I}^I - \dot{\theta}_i^I = 0$, $\dot{\theta}_{N^{II}}^{II} - \dot{\theta}_1^{II} = 0$, $\dot{\theta}_{N^{II}}^{II} - \dot{\theta}_m^{II} = 0$. Taking these into (S29), we have

$$
\sin\left(\theta_{N^I}^I - \theta_{N^{II}}^{II}\right) = \frac{\left(N^I - 2\right)\omega - \lambda\left(N^I - 1\right)\sin a - \lambda \sin c}{\beta}. \tag{S30}
$$

Since the r.h.s. of (S30) is a constant, $\sin\left(\theta_{N^I}^I - \theta_{N^{II}}^{II}\right)$ in the l.h.s. is a constant too. Consequently, $\dot{\theta}_1^I = \dot{\theta}_2^I = ... = \dot{\theta}_{N^I}^I = \dot{\theta}_1^{II} = \dot{\theta}_2^{II} = ... = \dot{\theta}_{N^{II}}^{II}$, the substitution of which into (S29) yields

$$
\sin\left(\theta_1^I - \theta_1^{II}\right) = \frac{-\lambda \sin a + \lambda \sin c}{\beta}, \tag{S31}
$$

$$
\sin c + \sin d = 2 \sin a, \tag{S32}
$$

$$
\sin a = \sin b = \frac{N^I + N^{II} - 4}{\lambda\left(N^I + N^{II}\right)}\omega. \tag{S33}
$$

Since $\theta_{N^I}^I - \theta_{N^{II}}^{II} = \left(\theta_{N^I}^I - \theta_1^I\right) + \theta_1^I - \theta_1^{II} + \left(\theta_1^{II} - \theta_{N^{II}}^{II}\right) = c - d + \theta_1^I - \theta_1^{II}$, we get $\beta \sin\left(c - d + \theta_1^I - \theta_1^{II}\right) = \left(N^I - 2\right)\omega - \lambda\left(N^I - 1\right)\sin a - \lambda \sin c$ from (S30), indicating that

$$
\arcsin \frac{\left(N^I - 2\right)\omega - \lambda\left(N^I - 1\right)\sin a - \lambda \sin c}{\beta} = c - \arcsin\left(2 \sin a - \sin c\right) + \arcsin \frac{-\lambda \sin a + \lambda \sin c}{\beta}. \tag{S34}
$$

By the setting of $x = \sin a - \sin c$, (S34) can be rearranged as

$$
\begin{aligned}
y_1(x) &= \arcsin\left(\sin a - x\right) - \arcsin\left(\sin a + x\right), \\
y_2(x) &= \arcsin\left[\frac{2\left(N^I - N^{II}\right)}{\beta\left(N^I + N^{II}\right)}\omega + \frac{\lambda}{\beta}x\right] + \arcsin\left(\frac{\lambda}{\beta}x\right).
\end{aligned}
\tag{S35}
$$

parameter \ endpoint	left endpoint x_1	right endpoint x_2
$\beta \geqslant \lambda - \frac{-N^I+3N^{II}-4}{N^I+N^{II}}\omega$	$-1 + \frac{N^I+N^{II}-4}{\lambda\left(N^I+N^{II}\right)}\omega$	$1 - \frac{N^I+N^{II}-4}{\lambda\left(N^I+N^{II}\right)}\omega$
$\lambda - \frac{N^I+N^{II}-4}{N^I+N^{II}}\omega \leqslant \beta < \lambda - \frac{-N^I+3N^{II}-4}{N^I+N^{II}}\omega$	$-1 + \frac{N^I+N^{II}-4}{\lambda\left(N^I+N^{II}\right)}\omega$	$\frac{\beta}{\lambda} - \frac{2\left(N^I-N^{II}\right)}{\lambda\left(N^I+N^{II}\right)}\omega$
$\beta < \lambda - \frac{N^I+N^{II}-4}{N^I+N^{II}}\omega$	$-\frac{\beta}{\lambda}$	$\frac{\beta}{\lambda} - \frac{2\left(N^I-N^{II}\right)}{\lambda\left(N^I+N^{II}\right)}\omega$

Table S1: The endpoints in **H-H L-L**. Here, we set $N^I \geqslant N^{II}$, w.l.o.g.

Obviously, if y_1 and y_2 in (S35) have intersection during the common domain of the two functions, (S34) has solution and the system can be synchronized. The common domain is determined by arcsin in (S35) since its domain is limited in $[-1, 1]$. Through the observation of simulation, we find that if $y_1 \geqslant y_2$ at the left endpoint of the domain and $y_1 \leqslant y_2$ at the right endpoint of the domain, y_1 and y_2 have intersection. Without loss of generation, we set $N^I \geqslant N^{II}$. And we record the left endpoint as x_1, while the right endpoint as x_2, same for case **H-L L-H** and case **H-L L-L**. In sum, there are two conditions for the synchronization:

1. $y_1(x_1) \geqslant y_2(x_1)$;
2. $y_1(x_2) \leqslant y_2(x_2)$.

The endpoints are based on the values of the coupling strengths and node numbers, seen as Table S1. Since the endpoints are divided into three groups, there are three groups of discussions as below.

1) $\beta \geqslant \lambda - \frac{-N^I+3N^{II}-4}{N^I+N^{II}}\omega$

With the left endpoint $x_1 = -1 + \frac{N^I+N^{II}-4}{\lambda\left(N^I+N^{II}\right)}\omega$, (S35) becomes

$$y_1\left(-1 + \frac{N^I + N^{II} - 4}{\lambda(N^I + N^{II})}\omega\right) = \frac{\pi}{2} - \arcsin\left[\frac{2\left(N^I + N^{II} - 4\right)}{\lambda(N^I + N^{II})}\omega - 1\right] \geqslant 0,$$
$$y_2\left(-1 + \frac{N^I + N^{II} - 4}{\lambda(N^I + N^{II})}\omega\right) = \arcsin\left[-\frac{\lambda}{\beta} + \frac{3N^I - N^{II} - 4}{\beta(N^I + N^{II})}\omega\right] + \arcsin\left[-\frac{\lambda}{\beta} + \frac{N^I + N^{II} - 4}{\beta(N^I + N^{II})}\omega\right]. \tag{S36}$$

If $\lambda \geqslant \frac{2\left(N^I-2\right)}{N^I+N^{II}}\omega$, $y_2\left(-1 + \frac{N^I+N^{II}-4}{N^I+N^{II}}\omega\right) \leqslant 0 \leqslant y_1\left(-1 + \frac{N^I+N^{II}-4}{N^I+N^{II}}\omega\right)$, which means that the first condition $y_1(x_1) \geqslant y_2(x_1)$ is satisfied. For $\lambda < \frac{2\left(N^I-2\right)}{N^I+N^{II}}\omega$, after the linearization of $y_1(x_1) \geqslant y_2(x_1)$, we have

$$\frac{\pi}{2} - \arcsin\left[\frac{2\left(N^I + N^{II} - 4\right)}{\lambda(N^I + N^{II})}\omega - 1\right] \geqslant 2\frac{-\lambda\left(N^I + N^{II}\right) + 2\left(N^I - 2\right)\omega}{\beta(N^I + N^{II})}, \tag{S37}$$

which yields

$$\beta \geqslant 2\frac{-\lambda\left(N^{I}+N^{II}\right)+2\left(N^{I}-2\right)\omega}{\left(N^{I}+N^{II}\right)\left\{\frac{\pi}{2}-\arcsin\left[\frac{2\left(N^{I}+N^{II}-4\right)}{\lambda\left(N^{I}+N^{II}\right)}\omega-1\right]\right\}}. \tag{S38}$$

As for the right endpoint $x_2 = 1-\frac{N^{I}+N^{II}-4}{N^{I}+N^{II}}\omega$, we have

$$\begin{aligned} y_1\left(1-\frac{N^{I}+N^{II}-4}{\lambda\left(N^{I}+N^{II}\right)}\omega\right) &= -\frac{\pi}{2}+\arcsin\left[\frac{2\left(N^{I}+N^{II}-4\right)}{\lambda\left(N^{I}+N^{II}\right)}\omega-1\right] \leqslant 0, \\ y_2\left(1-\frac{N^{I}+N^{II}-4}{\lambda\left(N^{I}+N^{II}\right)}\omega\right) &= \arcsin\left[\frac{\lambda}{\beta}+\frac{N^{I}-3N^{II}+4}{\beta\left(N^{I}+N^{II}\right)}\omega\right]+\arcsin\left[\frac{\lambda}{\beta}-\frac{N^{I}+N^{II}-4}{\beta\left(N^{I}+N^{II}\right)}\omega\right]. \end{aligned} \tag{S39}$$

Since $\lambda \geqslant \frac{2\left(N^{II}-2\right)}{N^{I}+N^{II}}\omega$, $y_2\left(1-\frac{N^{I}+N^{II}-4}{N^{I}+N^{II}}\omega\right) \geqslant 0 \geqslant y_1\left(1-\frac{N^{I}+N^{II}-4}{N^{I}+N^{II}}\omega\right)$. Thus, the second condition $y_1(x_2) \leqslant y_2(x_2)$ is satisfied without the limitation on the coupling strengths.

2) $\lambda-\frac{N^{I}+N^{II}-4}{N^{I}+N^{II}}\omega \leqslant \beta < \lambda-\frac{-N^{I}+3N^{II}-4}{N^{I}+N^{II}}\omega$

Here, since the left endpoint is as same as above, we get the same result as (S38) and only need to deal with the right endpoint. For the right endpoint $x_2 = \frac{\beta}{\lambda}-\frac{2\left(N^{I}-N^{II}\right)}{\lambda\left(N^{I}+N^{II}\right)}\omega$, we have

$$\begin{aligned} y_1\left(\frac{\beta}{\lambda}-\frac{2\left(N^{I}-N^{II}\right)}{\lambda\left(N^{I}+N^{II}\right)}\omega\right) &= \arcsin\left(-\frac{\beta}{\lambda}+\frac{3N^{I}-N^{II}-4}{\lambda\left(N^{I}+N^{II}\right)}\omega\right)+\arcsin\left(-\frac{\beta}{\lambda}+\frac{N^{I}-3N^{II}+4}{\lambda\left(N^{I}+N^{II}\right)}\omega\right), \\ y_2\left(\frac{\beta}{\lambda}-\frac{2\left(N^{I}-N^{II}\right)}{\lambda\left(N^{I}+N^{II}\right)}\omega\right) &= \frac{\pi}{2}+\arcsin\left[1-\frac{2\left(N^{I}-N^{II}\right)}{\beta\left(N^{I}+N^{II}\right)}\omega\right]. \end{aligned} \tag{S40}$$

The second condition $y_1\left(\frac{\beta}{\lambda}-\frac{2\left(N^{I}-N^{II}\right)}{\lambda\left(N^{I}+N^{II}\right)}\omega\right) \leqslant y_2\left(\frac{\beta}{\lambda}-\frac{2\left(N^{I}-N^{II}\right)}{\lambda\left(N^{I}+N^{II}\right)}\omega\right)$ yields

$$\arcsin\left(-\frac{\beta}{\lambda}+\frac{3N^{I}-N^{II}-4}{\lambda\left(N^{I}+N^{II}\right)}\omega\right)+\arcsin\left(-\frac{\beta}{\lambda}+\frac{N^{I}-3N^{II}+4}{\lambda\left(N^{I}+N^{II}\right)}\omega\right) \leqslant \frac{\pi}{2}+\arcsin\left[1-\frac{2\left(N^{I}-N^{II}\right)}{\beta\left(N^{I}+N^{II}\right)}\omega\right]. \tag{S41}$$

When λ is very large, the l.h.s. of (S41) equals to 0. Thus, the r.h.s. $\geqslant 0$, which leads to

$$\beta \geqslant \frac{N^{I}-N^{II}}{N^{I}+N^{II}}\omega. \tag{S42}$$

When λ is not very large, (S41) needs to be linearized and is reduced as

$$\frac{2}{\lambda}\beta^2+\left[\frac{\pi+2}{2}-\frac{4\left(N^{I}-N^{II}\right)}{\lambda\left(N^{I}+N^{II}\right)}\omega\right]-\frac{2\left(N^{I}-N^{II}\right)}{N^{I}+N^{II}}\omega \geqslant 0. \tag{S43}$$

Since (S43) is a quadratic equation with $\frac{2}{\lambda} > 0$ and $\triangle \geqslant 0$, we obtain the constrain on β as

$$\beta \geqslant -\frac{\pi+2}{8}\lambda + \frac{N^I - N^{II}}{N^I + N^{II}}\omega + \sqrt{\left(\frac{\pi+2}{8}\lambda - \frac{N^I - N^{II}}{N^I + N^{II}}\omega\right)^2 + \frac{\lambda\left(N^I - N^{II}\right)}{N^I + N^{II}}\omega} \tag{S44}$$

3) $\beta < \lambda - \frac{N^I + N^{II} - 4}{N^I + N^{II}}\omega$

Here, the right endpoint is the same as above and we can get the same result as (S44). For the left endpoint $x_1 = -\frac{\beta}{\lambda}$, we have

$$\begin{aligned} y_1\left(-\frac{\beta}{\lambda}\right) &= \arcsin\left(\frac{\beta}{\lambda} + \frac{N^I + N^{II} - 4}{\lambda\left(N^I + N^{II}\right)}\omega\right) + \arcsin\left(\frac{\beta}{\lambda} - \frac{N^I + N^{II} - 4}{\lambda\left(N^I + N^{II}\right)}\omega\right) > 0, \\ y_2\left(-\frac{\beta}{\lambda}\right) &= \arcsin\left[\frac{2\left(N^I - N^{II}\right)}{\beta\left(N^I + N^{II}\right)} - 1\right] - \frac{\pi}{2} \leqslant 0 \end{aligned} \tag{S45}$$

Since $y_1\left(-\frac{\beta}{\lambda}\right) \geqslant 0 \geqslant y_2\left(-\frac{\beta}{\lambda}\right)$, there is no limit on β for the second condition $y_1\left(x_1\right) \geqslant y_2\left(x_1\right)$.

Unlike the case in one-link connection, the two critical coupling strengths are inter-dependence. For union, we set the critical external coupling strength as a function of internal coupling strength, i.e. $\beta_c = f(\lambda)$. In sum, we have

$$\lambda^{\textbf{H-H L-L}} = \frac{N^I + N^{II} - 4}{N^I + N^{II}}\omega, \tag{S46}$$

$$\beta_c^{\textbf{H-H L-L}} = \max\begin{cases} 2\dfrac{-\lambda\left(N^I + N^{II}\right) + 2\left(\max\left\{N^I, N^{II}\right\} - 2\right)\omega}{\left(N^I + N^{II}\right)\left[\frac{\pi}{2} + \arcsin\left(1 - 2\frac{N^I + N^{II} - 4}{\lambda\left(N^I + N^{II}\right)}\omega\right)\right]} \\ -\dfrac{\pi+2}{8}\lambda + \dfrac{\left|N^I - N^{II}\right|}{N^I + N^{II}}\omega + \sqrt{\left(\dfrac{\pi+2}{8}\lambda - \dfrac{\left|N^I - N^{II}\right|}{N^I + N^{II}}\omega\right)^2 + \dfrac{\lambda\left|N^I - N^{II}\right|}{N^I + N^{II}}\omega} \end{cases}, \tag{S47}$$

$$\beta_{min}^{\textbf{H-H L-L}} = \frac{\left|N^I - N^{II}\right|}{N^I + N^{II}}\omega. \tag{S48}$$

S3.2 Case H-L L-H

The locking manifolds in case **H-L L-H** are as same as that in case **L-L** (S22). Using the locking manifolds (S22), (S2) provides

$$\begin{aligned}
&\dot{\theta}_1^I = \omega + \lambda \sin c - \beta \sin\left(\theta_1^I - \theta_{N^{II}}^{II}\right),\\
&\dot{\theta}_i^I = \omega + \lambda \sin a, i = 2, .., N^I - 1,\\
&\dot{\theta}_{N^I}^I = \left(N^I - 1\right)\omega - \lambda\left(N^I - 2\right)\sin a - \lambda \sin c - \beta \sin\left(\theta_{N^I}^I - \theta_1^{II}\right),\\
&\dot{\theta}_1^{II} = \omega + \lambda \sin d - \beta \sin\left(\theta_1^{II} - \theta_{N^I}^I\right),\\
&\dot{\theta}_m^{II} = \omega + \lambda \sin b, m = 2, .., N^{II} - 1,\\
&\dot{\theta}_{N^{II}}^{II} = \left(N^{II} - 1\right)\omega - \lambda\left(N^{II} - 2\right)\sin b - \lambda \sin d - \beta \sin\left(\theta_{N^{II}}^{II} - \theta_1^I\right).
\end{aligned} \tag{S49}$$

According to the locking manifolds, $\dot{\theta}_{N^I}^I - \dot{\theta}_1^I = 0$, $\dot{\theta}_{N^I}^I - \dot{\theta}_i^I = 0$, $\dot{\theta}_{N^{II}}^{II} - \dot{\theta}_1^{II} = 0$, $\dot{\theta}_{N^{II}}^{II} - \dot{\theta}_m^{II} = 0$. Taking these conditions into (S49), we have

$$\beta \sin\left(\theta_{N^{II}}^{II} - \theta_1^I\right) = \lambda \sin a - \lambda \sin c, \tag{S50}$$

$$\beta \sin\left(\theta_{N^I}^I - \theta_1^{II}\right) = \lambda \sin b - \lambda \sin d. \tag{S51}$$

Here, $\sin\left(\theta_{N^{II}}^{II} - \theta_1^I\right)$ and $\sin\left(\theta_{N^I}^I - \theta_1^{II}\right)$ are constants, which leads to $\dot{\theta}_{N^{II}}^{II} = \dot{\theta}_1^I$ and $\dot{\theta}_{N^I}^I = \dot{\theta}_1^{II}$. Consequently,

$$\dot{\theta}_1^I = \dot{\theta}_2^I = ... = \dot{\theta}_{N^I}^I = \dot{\theta}_1^{II} = \dot{\theta}_2^{II} = ... = \dot{\theta}_{N^{II}}^{II}. \tag{S52}$$

The substitution of (S52) into (S49) yields

$$\sin a = \sin b, \tag{S53}$$

$$\left(N^{II} - 2\right)\omega - \lambda\left(N^{II} - 2\right)\sin b - \lambda \sin c - \lambda \sin d - 2\beta \sin\left(\theta_{N^{II}}^{II} - \theta_1^I\right) = 0, \tag{S54}$$

$$\left(N^I - N^{II}\right)\omega - \lambda\left[\left(N^I - 2\right)\sin a - \left(N^{II} - 2\right)\sin b\right] = \lambda\left(\sin c - \sin d\right) + \beta\left[\sin\left(\theta_{N^I}^I - \theta_1^{II}\right) - \sin\left(\theta_{N^{II}}^{II} - \theta_1^I\right)\right]. \tag{S55}$$

The combination of (S50), (S53) and (S54) results in

$$\sin d - \sin c = \frac{\left(N^{II} - 2\right)\omega - \lambda N^{II} \sin a}{\lambda}. \tag{S56}$$

Similarly, combing (S50), (S51), (S53) and (S55) together, we have

$$\sin c - \sin d = \frac{\left(N^I - N^{II}\right)\omega - \lambda\left(N^I - N^{II}\right)\sin a}{2\lambda}. \tag{S57}$$

The addition of (S56) and (57) yields

$$\sin a = \frac{N^I + N^{II} - 4}{\lambda\left(N^I + N^{II}\right)}\omega. \tag{S58}$$

endpoint / parameter	left endpoint	right endpoint
$\beta \geqslant \lambda + \frac{N^I+N^{II}-4}{N^I+N^{II}}\omega$	$-1 + \frac{2(N^I-N^{II})}{\lambda(N^I+N^{II})}\omega$	1
$\lambda - \frac{N^I+N^{II}-4}{N^I+N^{II}}\omega \leqslant \beta < \lambda + \frac{N^I+N^{II}-4}{N^I+N^{II}}\omega$	$-\frac{\beta}{\lambda} + \frac{3N^I-N^{II}-4}{\lambda(N^I+N^{II})}\omega$	1
$\beta < \lambda - \frac{N^I+N^{II}-4}{N^I+N^{II}}\omega$	$-\frac{\beta}{\lambda} + \frac{3N^I-N^{II}-4}{\lambda(N^I+N^{II})}\omega$	$\frac{\beta}{\lambda} + \frac{N^I+N^{II}-4}{\lambda(N^I+N^{II})}\omega$

Table S2: The endpoints in **H-L L-H**. Here, we set $N^I \geqslant N^{II}$, w.l.o.g.

Since $\theta^{II}_{N^{II}} - \theta^I_1 = \left(\theta^{II}_{N^{II}} - \theta^{II}_1\right) + \theta^{II}_1 - \theta^I_{N^I} + \left(\theta^I_{N^I} - \theta^I_1\right) = d + c - \left(\theta^I_{N^I} - \theta^{II}_1\right)$, the combination of (S50) and (S51) leads to

$$\arcsin\frac{\lambda\sin a - \lambda\sin c}{\beta} = d + c - \arcsin\frac{\lambda\sin a - \lambda\sin d}{\beta}. \tag{S59}$$

Through (S57) and (S59), we obtain the transcendental equations

$$\begin{aligned} \sin d &= \sin c - \frac{\left(N^I - N^{II}\right)\omega - \lambda\left(N^I - N^{II}\right)\sin a}{2\lambda}, \\ d &= -c + \arcsin\frac{\lambda\sin a - \lambda\sin c}{\beta} + \arcsin\frac{\lambda\sin a - \lambda\sin d}{\beta}, \end{aligned} \tag{S60}$$

which can be rearranged as

$$\begin{aligned} y_1(\sin c) &= \arcsin(\sin c) + \arcsin\left[\sin c - \frac{2\left(N^I - N^{II}\right)}{\lambda\left(N^I + N^{II}\right)}\omega\right], \\ y_2(\sin c) &= \arcsin\frac{\lambda(\sin a - \sin c)}{\beta} + \arcsin\left[\frac{\lambda(\sin a - \sin c)}{\beta} + \frac{2\left(N^I - N^{II}\right)}{\beta\left(N^I + N^{II}\right)}\omega\right]. \end{aligned} \tag{S61}$$

After the observation of simulation, we find out the two conditions for the synchronization as

1. $y_1(x_1) \leqslant y_2(x_1)$;
2. $y_1(x_2) \geqslant y_2(x_2)$.

The endpoints of the common domain of y_1 and y_2 can be seen in Table. S2. According to the different values of endpoints, there are three groups of discussions in the following part. Without loss of generation, we set $N^I \geqslant^{II}$.

1) $\beta \geqslant \lambda + \frac{N^I+N^{II}-4}{N^I+N^{II}}\omega$

For the left endpoint $x_1 = -1 + \frac{2\left(N^I - N^{II}\right)}{\lambda\left(N^I + N^{II}\right)}\omega$, we have

$$
\begin{aligned}
y_1\left(-1 + \frac{2\left(N^I - N^{II}\right)}{\lambda\left(N^I + N^{II}\right)}\omega\right) &= \arcsin\left(-1 + + \frac{2\left(N^I - N^{II}\right)}{\lambda\left(N^I + N^{II}\right)}\omega\right) - \frac{\pi}{2} \leqslant 0, \\
y_2\left(-1 + \frac{2\left(N^I - N^{II}\right)}{\lambda\left(N^I + N^{II}\right)}\omega\right) &= \arcsin\left(\frac{\lambda}{\beta} + \frac{-N^I + 3N^{II} - 4}{\beta\left(N^I + N^{II}\right)}\right) + \arcsin\left(\frac{\lambda}{\beta} + \frac{N^I + N^{II} - 4}{\beta\left(N^I + N^{II}\right)}\right).
\end{aligned}
\tag{S62}
$$

Since $\lambda \geqslant \frac{N^I + N^{II} - 4}{N^I + N^{II}}\omega$, $y_2\left(-1 + \frac{2\left(N^I - N^{II}\right)}{\lambda\left(N^I + N^{II}\right)}\omega\right) \geqslant 0 \geqslant y_1\left(-1 + \frac{2\left(N^I - N^{II}\right)}{\lambda\left(N^I + N^{II}\right)}\omega\right)$, which means the first condition $y_1\left(x_1\right) \leqslant y_2\left(x_1\right)$ is satisfied without limitation.

For the right endpoint $x_2 = 1$, we have

$$
\begin{aligned}
y_1(1) &= \frac{\pi}{2} + \arcsin\left[1 - \frac{2\left(N^I - N^{II}\right)}{\lambda\left(N^I + N^{II}\right)}\omega\right] \geqslant 0, \\
y_2(1) &= \arcsin\left[-\frac{\lambda}{\beta} + \frac{N^I + N^{II} - 4}{\beta\left(N^I + N^{II}\right)}\omega\right] + \arcsin\left[-\frac{\lambda}{\beta} + \frac{3N^I - N^{II} - 4}{\beta\left(N^I + N^{II}\right)}\omega\right].
\end{aligned}
\tag{S63}
$$

If $\lambda \geqslant \frac{2\left(N^I - 2\right)}{N^I + N^{II}}\omega$, $y_2(1) \leqslant 0 \leqslant y_1(1)$. When $\lambda \leqslant \frac{2\left(N^I - 2\right)}{N^I + N^{II}}\omega$, the linearization of $y_1(1) \geqslant y_2(1)$ from (S63) is needed and leads to

$$
\frac{\pi}{2} + \arcsin\left[1 - \frac{2\left(N^I - N^{II}\right)}{\lambda\left(N^I + N^{II}\right)}\omega\right] \geqslant 2\frac{-\lambda\left(N^I + N^{II}\right) + 2\left(N^I - 2\right)\omega}{\beta\left(N^I + N^{II}\right)}. \tag{S64}
$$

Namely,

$$
\beta \geqslant 2\frac{-\lambda\left(N^I + N^{II}\right) + 2\left(N^I - 2\right)\omega}{\left(N^I + N^{II}\right)\left\{\frac{\pi}{2} + \arcsin\left[1 - \frac{2\left(N^I - N^{II}\right)}{\lambda\left(N^I + N^{II}\right)}\omega\right]\right\}}. \tag{S65}
$$

2) $\lambda - \frac{N^I + N^{II} - 4}{N^I + N^{II}}\omega \leqslant \beta < \lambda + \frac{N^I + N^{II} - 4}{N^I + N^{II}}\omega$

For the left endpoint $x_1 = -\frac{\beta}{\lambda} + \frac{3N^I - N^{II} - 4}{\lambda\left(N^I + N^{II}\right)}\omega$, we have

$$
\begin{aligned}
y_1\left(-\frac{\beta}{\lambda} + \frac{3N^I - N^{II} - 4}{\lambda\left(N^I + N^{II}\right)}\omega\right) &= \arcsin\left[-\frac{\beta}{\lambda} + \frac{3N^I - N^{II} - 4}{\lambda\left(N^I + N^{II}\right)}\omega\right] z + \arcsin\left[-\frac{\beta}{\lambda} + \frac{N^I + N^{II} - 4}{\lambda\left(N^I + N^{II}\right)}\omega\right], \\
y_2\left(-\frac{\beta}{\lambda} + \frac{3N^I - N^{II} - 4}{\lambda\left(N^I + N^{II}\right)}\omega\right) &= \arcsin\left[1 - \frac{2\left(N^I - N^{II}\right)}{\beta\left(N^I + N^{II}\right)}\omega\right] + \frac{\pi}{2}.
\end{aligned}
\tag{S66}
$$

Since $1 - \frac{2\left(N^I - N^{II}\right)}{\beta\left(N^I + N^{II}\right)}\omega \geqslant -1$, we obtain

$$
\beta \geqslant \frac{N^I - N^{II}}{N^I + N^{II}}\omega \tag{S67}
$$

The first condition $y_1\left(-\frac{\beta}{\lambda}+\frac{3N^I-N^{II}-4}{\lambda\left(N^I+N^{II}\right)}\omega\right)\leqslant y_2\left(-\frac{\beta}{\lambda}+\frac{3N^I-N^{II}-4}{\lambda\left(N^I+N^{II}\right)}\omega\right)$ means

$$\arcsin\left[-\frac{\beta}{\lambda}+\frac{3N^I-N^{II}-4}{\lambda\left(N^I+N^{II}\right)}\omega\right]+\arcsin\left[-\frac{\beta}{\lambda}+\frac{N^I+N^{II}-4}{\lambda\left(N^I+N^{II}\right)}\omega\right]\leqslant\arcsin\left[1-\frac{2\left(N^I-N^{II}\right)}{\beta\left(N^I+N^{II}\right)}\omega\right]+\frac{\pi}{2}. \tag{S68}$$

The linearization of (S68) yields

$$\frac{2}{\lambda}\beta^2+\left[\frac{\pi+2}{2}-\frac{4\left(N^I-2\right)}{\lambda\left(N^I+N^{II}\right)}\omega\right]+\frac{2\left(N^I-N^{II}\right)}{N^I+N^{II}}\omega\geqslant 0. \tag{S69}$$

Since (S69) is a quadratic equation with $\frac{2}{\lambda}>0$ and $\triangle\geqslant 0$, we have

$$\beta\geqslant-\frac{\pi+2}{8}\lambda+\frac{N^I-2}{N^I+N^{II}}\omega+\sqrt{\left(\frac{\pi+2}{8}\lambda-\frac{N^I-2}{N^I+N^{II}}\omega\right)^2+\frac{\lambda\left(N^I-N^{II}\right)}{N^I+N^{II}}\omega}. \tag{S70}$$

The requirement brought by the second condition here is as same as (S65).

3) $\beta<\lambda-\frac{N^I+N^{II}-4}{N^I+N^{II}}\omega$

Owing to the same left endpoint, the requirement brought by the first condition $y_1\left(x_1\right)\leqslant y_2\left(x_1\right)$ is the same as (S70). As for the right endpoint $x_2=\frac{\beta}{\lambda}+\frac{N^I+N^{II}-4}{\lambda\left(N^I+N^{II}\right)}\omega$, we have

$$\begin{aligned}y_1\left(\frac{\beta}{\lambda}+\frac{N^I+N^{II}-4}{\lambda\left(N^I+N^{II}\right)}\omega\right)&=\arcsin\left[\frac{\beta}{\lambda}+\frac{N^I+N^{II}-4}{N^I+N^{II}}\omega\right]+\arcsin\left[\frac{\beta}{\lambda}+\frac{-N^I+3N^{II}-4}{N^I+N^{II}}\omega\right],\\ y_2\left(\frac{\beta}{\lambda}+\frac{N^I+N^{II}-4}{\lambda\left(N^I+N^{II}\right)}\omega\right)&=-\frac{\pi}{2}+\arcsin\left[-1+\frac{2\left(N^I-N^{II}\right)}{\beta\left(N^I+N^{II}\right)}\omega\right]\leqslant 0.\end{aligned} \tag{S71}$$

Since $\frac{\beta}{\lambda}+\frac{N^I+N^{II}-4}{N^I+N^{II}}\omega+\frac{\beta}{\lambda}+\frac{-N^I+3N^{II}-4}{N^I+N^{II}}\omega>0$, $y_1\left(\frac{\beta}{\lambda}+\frac{N^I+N^{II}-4}{\lambda\left(N^I+N^{II}\right)}\omega\right)>0\geqslant y_2\left(\frac{\beta}{\lambda}+\frac{N^I+N^{II}-4}{\lambda\left(N^I+N^{II}\right)}\omega\right)$, indicating that the second condition $y_1\left(x_2\right)\geqslant y_2\left(x_2\right)$ is satisfied.

Similar to case **H-H L-L**, we set β_c as a function of λ and get

$$\lambda^{\textbf{H-L L-H}}=\frac{N^I+N^{II}-4}{N^I+N^{II}}\omega, \tag{S72}$$

$$\beta_c^{\textbf{H-L L-H}}=\max\begin{cases}2\dfrac{-\lambda\left(N^I+N^{II}\right)+2\left(\max\left\{N^I,N^{II}\right\}-2\right)\omega}{\left(N^I+N^{II}\right)\left\{\frac{\pi}{2}+\arcsin\left[1-\frac{2\left|N^I-N^{II}\right|}{\lambda\left(N^I+N^{II}\right)}\omega\right]\right\}}\\ -\dfrac{\pi+2}{8}\lambda+\dfrac{N^I-2}{N^I+N^{II}}\omega+\sqrt{\left(\dfrac{\pi+2}{8}\lambda-\dfrac{N^I-2}{N^I+N^{II}}\omega\right)^2+\dfrac{\lambda\left|N^I-N^{II}\right|}{N^I+N^{II}}\omega}\end{cases}, \tag{S73}$$

$$\beta_{min}^{\textbf{H-L L-H}}=\frac{\left|N^I-N^{II}\right|}{N^I+N^{II}}\omega. \tag{S74}$$

S3.3 Case L-L L-L

The locking manifolds for two synchronized networks in **L-L L-L** case are

$$
\begin{aligned}
\mathrm{M}_1 &= \left\{\theta^I_{N^I} - \theta^I_i = a, i = 2, ..., N^I - 1, a \in [0, 2\pi)\right\},\\
\mathrm{M}_2 &= \left\{\theta^{II}_{N^{II}} - \theta^{II}_m = b, m = 2, ..., N^{II} - 1, b \in [0, 2\pi)\right\},\\
\mathrm{M}_3 &= \left\{\theta^I_{N^I} - \theta^I_1 = c, c \in [0, 2\pi)\right\},\\
\mathrm{M}_4 &= \left\{\theta^{II}_{N^{II}} - \theta^{II}_1 = d, d \in [0, 2\pi)\right\},\\
\mathrm{M}_5 &= \left\{\theta^I_{N^I} - \theta^I_2 = e, e \in [0, 2\pi)\right\},\\
\mathrm{M}_6 &= \left\{\theta^{II}_{N^{II}} - \theta^{II}_2 = f, f \in [0, 2\pi)\right\}.
\end{aligned}
\tag{S75}
$$

Here, the leaf node 1^I connects to the leaf node 1^{II} and the leaf node 2^I connects to the leaf node 2^{II}. The dynamical equations for the synchronized system are

$$
\begin{aligned}
\dot{\theta}^I_1 &= \omega + \lambda \sin c - \beta \sin\left(\theta^I_1 - \theta^{II}_1\right),\\
\dot{\theta}^I_2 &= \omega + \lambda \sin e - \beta \sin\left(\theta^I_2 - \theta^{II}_2\right),\\
\dot{\theta}^I_i &= \omega + \lambda \sin a, i = 3, ..., N^I - 1,\\
\dot{\theta}^I_{N^I} &= \left(N^I - 1\right)\omega - \left(N^I - 3\right)\lambda \sin a - \lambda \sin c - \lambda \sin e,\\
\dot{\theta}^{II}_1 &= \omega + \lambda \sin d + \beta \sin\left(\theta^I_1 - \theta^{II}_1\right),\\
\dot{\theta}^{II}_2 &= \omega + \lambda \sin f + \beta \sin\left(\theta^I_2 - \theta^{II}_2\right),\\
\dot{\theta}^{II}_m &= \omega + \lambda \sin b, m = 3, ..., N^{II} - 1,\\
\dot{\theta}^{II}_{N^{II}} &= \left(N^{II} - 1\right)\omega - \left(N^{II} - 3\right)\lambda \sin b - \lambda \sin d - \lambda \sin f.
\end{aligned}
\tag{S76}
$$

According to the locking manifolds, we get $\dot{\theta}^I_1 = \dot{\theta}^I_2 = \dot{\theta}^I_3 = ... = \dot{\theta}^I_{N^I} = \dot{\theta}^{II}_1 = \dot{\theta}^{II}_2 = \dot{\theta}^{II}_3 = ... = \dot{\theta}^{II}_{N^{II}}$ straightly, accompanying with which (S76) leads to

$$\sin a = \sin b = \frac{N^I + N^{II} - 4}{\lambda\left(N^I + N^{II}\right)}\omega, \tag{S77}$$

$$\beta \sin\left(\theta^I_1 - \theta^{II}_1\right) = \lambda \sin c - \lambda \sin a, \tag{S78}$$

$$\beta \sin\left(\theta^I_2 - \theta^{II}_2\right) = \lambda \sin e - \lambda \sin b. \tag{S79}$$

$$\sin c + \sin d = 2 \sin a, \tag{S80}$$

$$\sin e + \sin f = 2 \sin b, \tag{S81}$$

$$\left(N^I - 2\right)\omega - \left(N^I - 2\right)\lambda \sin a - \lambda \sin c - \lambda \sin e = 0, \tag{S82}$$

$$\left(N^{II} - 2\right)\omega - \left(N^{II} - 2\right)\lambda \sin b - \lambda \sin d - \lambda \sin f = 0. \tag{S83}$$

Using $\theta_1^I - \theta_1^{II} = \left(\theta_1^I - \theta_{N^I}^I\right) + \left(\theta_{N^I}^I - \theta_2^I\right) + \left(\theta_2^I - \theta_2^{II}\right) + \left(\theta_2^{II} - \theta_{N^{II}}^{II}\right) + \left(\theta_{N^{II}}^{II} - \theta_1^{II}\right)$, we apply Taylor approximation $\sin x = x + O(x)$ to (S78) with, and get

$$\beta\left[-c + e + \left(\theta_2^I - \theta_2^{II}\right) - f + d\right] = \lambda c - \lambda a. \tag{S84}$$

With the application of Taylor approximation $\sin x = x + O(x)$ to (S78) - (S81), the conclusion can be drawn as

$$c = e, d = f. \tag{S85}$$

It is noticed that the conclusion $c = \theta_{N^I}^I - \theta_1^I = \theta_{N^I}^I - \theta_2^I = e$ can be seen straightly if the initial phases differences of $\theta_{N^I}^I - \theta_1^I$ and $\theta_{N^I}^I - \theta_2^I$ are the same, since the dynamics of $\theta_{N^I}^I - \theta_1^I$ and $\theta_{N^I}^I - \theta_2^I$ are the same. Same for the conclusion $d = f$. Substituting $c = e, d = f$ into (S82) and (S83), we have

$$\sin c = \sin e = \frac{2\left(N^I - 2\right)}{\lambda\left(N^I + N^{II}\right)}\omega, \tag{S86}$$

$$\sin d = \sin f = \frac{2\left(N^{II} - 2\right)}{\lambda\left(N^I + N^{II}\right)}\omega. \tag{S87}$$

Thus, (S80) and (S81) lead to

$$\sin\left(\theta_{N^I}^I - \theta_1^{II}\right) = \frac{N^I - N^{II}}{\beta\left(N^I + N^{II}\right)}\omega, \tag{S88}$$

$$\sin\left(\theta_{N^{II}}^{II} - \theta_1^I\right) = \frac{N^{II} - N^I}{\beta\left(N^I + N^{II}\right)}\omega. \tag{S89}$$

Since the sine value is limited in $[-1, 1]$, the r.h.s. of (S77) and (S86-S89) result in

$$\lambda \geqslant \frac{N^I + N^{II} - 4}{N^I + N^{II}}\omega, \tag{S90}$$

$$\lambda \geqslant \frac{2\left(N^I - 2\right)}{N^I + N^{II}}\omega, \tag{S91}$$

$$\lambda \geqslant \frac{2\left(N^{II} - 2\right)}{N^I + N^{II}}\omega, \tag{S92}$$

$$\beta \geqslant \frac{\left|N^I - N^{II}\right|}{N^I + N^{II}}\omega. \tag{S93}$$

Consequently, the critical coupling strengths are:

$$\lambda_c^{\textbf{L-L L-L}} = \max\begin{cases} \dfrac{2N^I - 4}{N^I + N^{II}}\omega, & N^I \geqslant N^{II} \\ \dfrac{2N^{II} - 4}{N^I + N^{II}}\omega, & N^I < N^{II} \end{cases}, \tag{S94}$$

$$\beta_c^{\textbf{L-L L-L}} = \frac{\left|N^I - N^{II}\right|}{N^I + N^{II}}\omega. \tag{S95}$$

S3.4 Case H-L L-L

The locking manifolds for two synchronized networks in **H-L L-L** case are

$$\begin{aligned}
\mathrm{M}_1 &= \left\{\theta^I_{N^I} - \theta^I_i = a, i = 2, ..., N^I - 1, a \in [0, 2\pi)\right\},\\
\mathrm{M}_2 &= \left\{\theta^{II}_{N^{II}} - \theta^{II}_m = b, m = 3, ..., N^{II} - 1, b \in [0, 2\pi)\right\},\\
\mathrm{M}_3 &= \left\{\theta^I_{N^I} - \theta^I_1 = c, c \in [0, 2\pi)\right\},\\
\mathrm{M}_4 &= \left\{\theta^{II}_{N^{II}} - \theta^{II}_1 = d, d \in [0, 2\pi)\right\},\\
\mathrm{M}_5 &= \left\{\theta^{II}_{N^{II}} - \theta^{II}_2 = f, f \in [0, 2\pi)\right\}.
\end{aligned} \tag{S96}$$

Here, the hub node N^I connects to the leaf node 1^{II} and the leaf node 1^I connects to the leaf node θ^{II}_2. For the synchronized system, the dynamical equations (S2) can be rewritten as

$$\begin{aligned}
\dot\theta^I_1 &= \omega + \lambda \sin c - \beta \sin\left(\theta^I_1 - \theta^{II}_2\right),\\
\dot\theta^I_i &= \omega + \lambda \sin a, i = 2, ..., N^I - 1,\\
\dot\theta^I_{N^I} &= \left(N^I - 1\right)\omega - \left(N^I - 2\right)\lambda \sin a - \lambda \sin c - \beta \sin\left(\theta^I_{N^I} - \theta^{II}_1\right),\\
\dot\theta^{II}_1 &= \omega + \lambda \sin d + \beta \sin\left(\theta^I_{N^I} - \theta^{II}_1\right),\\
\dot\theta^{II}_2 &= \omega + \lambda \sin f + \beta \sin\left(\theta^I_1 - \theta^{II}_2\right),\\
\dot\theta^{II}_m &= \omega + \lambda \sin b, m = 3, ..., N^{II} - 1,\\
\dot\theta^{II}_{N^{II}} &= \left(N^{II} - 1\right)\omega - \left(N^{II} - 3\right)\lambda \sin b - \lambda \sin d - \lambda \sin f.
\end{aligned} \tag{S97}$$

Seen from (S97), we can draw the conclusion $\dot\theta^I_1 = \dot\theta^I_2 = \dot\theta^I_3 = ... = \dot\theta^I_{N^I} = \dot\theta^{II}_1 = \dot\theta^{II}_2 = \dot\theta^{II}_3 = ... = \dot\theta^{II}_{N^{II}}$ straightly. The substitution of this conclusion back into (S97) yields

$$\sin a = \sin b, \tag{S98}$$

$$\beta \sin\left(\theta^I_1 - \theta^{II}_2\right) = \lambda \sin c - \lambda \sin a, \tag{S99}$$

$$\beta \sin\left(\theta^I_{N^I} - \theta^{II}_1\right) = \lambda \sin a - \lambda \sin d. \tag{S100}$$

$$\beta \sin\left(\theta^I_{N^I} - \theta^{II}_1\right) = \left(N^I - 2\right)\omega - \left(N^I - 1\right)\lambda \sin a - \lambda \sin c; \tag{S101}$$

$$\left(N^{II} - 2\right)\omega - \left(N^{II} - 2\right)\lambda \sin a - \lambda \sin d - \lambda \sin f = 0, \tag{S102}$$

$$\sin c + \sin f = 2 \sin a. \tag{S103}$$

Substituting (S100) into (S101), we get

$$\sin c - \sin d = \frac{N^I - 2}{\lambda}\omega - N^I \sin a. \tag{S104}$$

The combination of (S102), (S103) and (S104) yields

$$\sin a = \frac{N^I + N^{II} - 4}{\lambda\left(N^I + N^{II}\right)}\omega, \tag{S105}$$

indicating that $\lambda \geq \frac{N^I+N^{II}-4}{\left(N^I+N^{II}\right)}\omega$. Since $\theta_1^I - \theta_2^{II} = -\left(\theta_{N^I}^I - \theta_1^I\right) + \left(\theta_{N^I}^I - \theta_1^{II}\right) - \left(\theta_{N^{II}}^{II} - \theta_1^{II}\right) + \left(\theta_{N^{II}}^{II} - \theta_2^{II}\right) = -c + \left(\theta_{N^I}^I - \theta_1^{II}\right) - d + f$, we obtain the following equation with (S99) and (S100):

$$\arcsin\frac{\lambda\sin c - \lambda\sin a}{\beta} = -c + \arcsin\frac{\lambda\sin a - \lambda\sin d}{\beta} - d + f. \tag{S106}$$

Using (S103), (S104) and (S106) jointly, the transcendental equations become

$$\begin{aligned}
&\sin f = 2\sin a - \sin c,\\
&\sin d = N^I\sin a + \sin c - \frac{N^I-2}{\lambda}\omega,\\
&\arcsin\frac{\lambda\sin c - \lambda\sin a}{\beta} = -c + \arcsin\frac{\lambda\sin a - \lambda\sin d}{\beta} - d + f,
\end{aligned} \tag{S107}$$

which require $2\sin a - \sin c \in [-1,1]$, $N^I\sin a + \sin c - \frac{N^I-2}{\lambda}\omega \in [-1,1]$, $\frac{\lambda\sin c - \lambda\sin a}{\beta} \in [-1,1]$ and $\frac{\lambda\sin a - \lambda\sin d}{\beta} \in [-1,1]$. Namely,

$$-1 - N^I\sin a + \frac{N^I-2}{\lambda}\omega \leqslant \sin c \leqslant 1 - N^I\sin a + \frac{N^I-2}{\lambda}\omega, \tag{S108}$$

$$-1 + 2\sin a \leqslant \sin c \leqslant 1 + 2\sin a, \tag{S109}$$

$$-\frac{\beta}{\lambda} - \left(N^I-1\right)\sin a + \frac{N^I-2}{\lambda}\omega \leqslant \sin c \leqslant \frac{\beta}{\lambda} - \left(N^I-1\right)\sin a + \frac{N^I-2}{\lambda}\omega, \tag{S110}$$

$$\frac{\beta}{\lambda} + \sin a \leqslant \sin c \leqslant \frac{\beta}{\lambda} + \sin a. \tag{S111}$$

The transcendental equations can be separated into two parts:

$$\begin{aligned}
&y_1(\sin c) = \arcsin(\sin c) + \arcsin\left[\sin c + N^I\sin a - \frac{N^I-2}{\lambda}\omega\right] - \arcsin(2\sin a - \sin c);\\
&y_2(\sin c) = \arcsin\frac{-\lambda\left(N^I-1\right)\sin a - \lambda\sin c + \left(N^I-2\right)\omega}{\beta} + \arcsin\frac{\lambda(\sin a - \sin c)}{\beta}.
\end{aligned} \tag{S112}$$

The system can be synchronized when (S107) has a solution, which means y_1 and y_2 in (S112) have intersection during the common domain. Through the observation of simulation, there are two conditions for the intersection:

1. $y_1(x_1) \leqslant y_2(x_1)$;
2. $y_1(x_2) \geqslant y_2(x_2)$.

The endpoints of the common domain of y_1 and y_2 are based on the values of the coupling strengths and node numbers, seen in Table. S3. There are three groups of discussions:

parameter \ endpoint		left endpoint x_1	right endpoint x_2
$N^I \geqslant N^{II}$	$\beta \geqslant \lambda - \frac{-N^I+3N^{II}-4}{N^I+N^{II}}\omega$	$-1 + \frac{2\left(N^I+N^{II}-4\right)}{\lambda\left(N^I+N^{II}\right)}\omega$	1
	$\lambda - \frac{N^I+N^{II}-4}{N^I+N^{II}}\omega \leqslant \beta < \lambda - \frac{-N^I+3N^{II}-4}{N^I+N^{II}}\omega$	$-\frac{\beta}{\lambda} + \frac{3N^I-N^{II}-4}{\lambda\left(N^I+N^{II}\right)}\omega$	1
	$\beta < \lambda - \frac{N^I+N^{II}-4}{N^I+N^{II}}\omega$	$-\frac{\beta}{\lambda} + \frac{3N^I-N^{II}-4}{\lambda\left(N^I+N^{II}\right)}\omega$	$\frac{\beta}{\lambda} + \frac{N^I+N^{II}-4}{\lambda\left(N^I+N^{II}\right)}\omega$
$N^I < N^{II}$	$\beta \geqslant \lambda - \frac{N^I+N^{II}-4}{N^I+N^{II}}\omega$	$-1 + \frac{2\left(N^I+N^{II}-4\right)}{\lambda\left(N^I+N^{II}\right)}\omega$	$1 - \frac{2\left(N^{II}-N^I\right)}{\lambda\left(N^I+N^{II}\right)}\omega$
	$\beta < \lambda - \frac{N^I+N^{II}-4}{N^I+N^{II}}\omega$	$-\frac{\beta}{\lambda} + \frac{N^I+N^{II}-4}{\lambda\left(N^I+N^{II}\right)}\omega$	$\frac{\beta}{\lambda} + \frac{3N^I-N^{II}-4}{\lambda\left(N^I+N^{II}\right)}\omega$

Table S3: The endpoints in **H-L L-L**.

1) $N^I \geqslant N^{II}, \beta \geqslant \lambda - \frac{-N^I+3N^{II}-4}{N^I+N^{II}}\omega$

For the left endpoint $x_1 = -1 + \frac{2\left(N^I+N^{II}-4\right)}{\lambda\left(N^I+N^{II}\right)}\omega$, (S112) turns into

$$
\begin{aligned}
y_1\left(-1 + \frac{2\left(N^I + N^{II} - 4\right)}{\lambda\left(N^I + N^{II}\right)}\omega\right) &= \arcsin\left[-1 + \frac{2\left(N^I + N^{II} - 4\right)}{\lambda\left(N^I + N^{II}\right)}\omega\right] + \arcsin\left[-1 + \frac{4\left(N^{II} - 2\right)}{\lambda\left(N^I + N^{II}\right)}\omega\right] - \frac{\pi}{2}, \\
y_2\left(-1 + \frac{2\left(N^I + N^{II} - 4\right)}{\lambda\left(N^I + N^{II}\right)}\omega\right) &= \arcsin\frac{\lambda - \frac{-N^I+3N^{II}-4}{N^I+N^{II}}\omega}{\beta} + \arcsin\frac{\lambda - \frac{N^I+N^{II}-4}{N^I+N^{II}}\omega}{\beta}.
\end{aligned}
\tag{S113}
$$

The linearization of the first condition $y_1(x_1) \leqslant y_2(x_1)$ is

$$
\arcsin\left[-1 + \frac{2\left(N^I + N^{II} - 4\right)}{\lambda\left(N^I + N^{II}\right)}\omega\right] + \arcsin\left[-1 + \frac{4\left(N^{II} - 2\right)}{\lambda\left(N^I + N^{II}\right)}\omega\right] - \frac{\pi}{2} \leqslant \frac{2\lambda\left(N^I + N^{II}\right) - 4\left(N^{II} - 2\right)\omega}{\beta\left(N^I + N^{II}\right)}.
\tag{S114}
$$

Since $\lambda \geqslant \frac{N^I+N^{II}-4}{N^I+N^{II}}$ and $N^I \geqslant N^{II}$, the r.h.s. of (S114) is larger than zero. Thus, if the l.h.s. of (S114) is less than zero, (S114) is satisfied. Setting the l.h.s. of (S114) as zero, we have $-1 + \frac{2\left(N^I+N^{II}-4\right)}{\lambda\left(N^I+N^{II}\right)}\omega > 0$, $-1 + \frac{4\left(N^{II}-2\right)}{\lambda\left(N^I+N^{II}\right)}\omega > 0$ and $\left[-1 + \frac{2\left(N^I+N^{II}-4\right)}{\lambda\left(N^I+N^{II}\right)}\omega\right]^2 + \left[-1 + \frac{4\left(N^{II}-2\right)}{\lambda\left(N^I+N^{II}\right)}\omega\right]^2 = 1$, which yield

$$
\lambda = 2\frac{N^I + 3N^{II} - 8 - 2\sqrt{(N^I + N^{II} - 4)(N^{II} - 2)}}{N^I + N^{II}}\omega.
\tag{S115}
$$

Hence, when $\lambda < 2\frac{N^I+3N^{II}-8-2\sqrt{(N^I+N^{II}-4)(N^{II}-2)}}{N^I+N^{II}}\omega$, there exits the maximal value of external coupling strength

$$
\beta \leqslant 2\frac{\lambda\left(N^I + N^{II}\right) - 2\left(N^{II} - 2\right)\omega}{(N^I + N^{II})\left\{\arcsin\left[\frac{2\left(N^I+N^{II}-4\right)}{\lambda\left(N^I+N^{II}\right)}\omega - 1\right] + \arcsin\left[\frac{4\left(N^{II}-2\right)}{\lambda\left(N^I+N^{II}\right)}\omega - 1\right] - \frac{\pi}{2}\right\}}.
\tag{S116}
$$

As for the right endpoint $x_2 = 1$, (S112) becomes

$$y_1(1) = \arcsin(1) + \arcsin\left[1 - \frac{2\left(N^I - N^{II}\right)}{\lambda(N^I + N^{II})}\right] + \arcsin\left[1 - \frac{2\left(N^I + N^{II} - 4\right)}{\lambda(N^I + N^{II})}\right],$$
$$y_2(1) = \arcsin\frac{-\lambda + \frac{3N^I - N^{II} - 4}{N^I + N^{II}}\omega}{\beta} + \arcsin\frac{-\lambda + \frac{N^I + N^{II} - 4}{N^I + N^{II}}\omega}{\beta}. \tag{S117}$$

To find out the requirement that satisfies the second condition $y_1(1) \geqslant y_2(1)$, the linearization of $y_1(1) \geqslant y_2(1)$ is needed and provides

$$\frac{\pi}{2} + \arcsin\left[1 - \frac{2\left(N^I - N^{II}\right)}{\lambda(N^I + N^{II})}\omega\right] + \arcsin\left[1 - \frac{2\left(N^I + N^{II} - 4\right)}{\lambda(N^I + N^{II})}\omega\right] \geqslant 2\frac{-\lambda\left(N^I + N^{II}\right) + 2\left(N^I - 2\right)\omega}{\beta(N^I + N^{II})}. \tag{S118}$$

It can be expressed as

$$\frac{\pi}{2} - \left\{\arcsin\left[\frac{2\left(N^I - N^{II}\right)}{\lambda(N^I + N^{II})}\omega - 1\right] + \arcsin\left[\frac{2\left(N^I + N^{II} - 4\right)}{\lambda(N^I + N^{II})}\omega - 1\right]\right\} \geqslant 2\frac{-\lambda\left(N^I + N^{II}\right) + 2\left(N^I - 2\right)\omega}{\beta(N^I + N^{II})}.$$

If the left side is less than 0 while the right side is larger than 0, than (S118) can never be found. That is $\lambda < \frac{2\left(N^I - 2\right)}{N^I + N^{II}}\omega$ and $\arcsin\left[\frac{2\left(N^I - N^{II}\right)}{\lambda\left(N^I + N^{II}\right)}\omega - 1\right] + \arcsin\left[\frac{2\left(N^I + N^{II} - 4\right)}{\lambda\left(N^I + N^{II}\right)}\omega - 1\right] > \frac{\pi}{2}$. The later requires $\arcsin\left[\frac{2\left(N^I - N^{II}\right)}{\lambda\left(N^I + N^{II}\right)}\omega - 1\right] > 0$ and $\arcsin\left[\frac{2\left(N^I + N^{II} - 4\right)}{\lambda\left(N^I + N^{II}\right)}\omega - 1\right] > 0$, meaning that $\lambda < \frac{2\left(N^I - N^{II}\right)}{\left(N^I + N^{II}\right)}\omega$ and $\lambda < \frac{2\left(N^I + N^{II} - 4\right)}{\lambda\left(N^I + N^{II}\right)}\omega$. The solution of the equation $\arcsin\left[\frac{2\left(N^I - N^{II}\right)}{\lambda\left(N^I + N^{II}\right)}\omega - 1\right] + \arcsin\left[\frac{2\left(N^I + N^{II} - 4\right)}{\lambda\left(N^I + N^{II}\right)}\omega - 1\right] = \frac{\pi}{2}$ is

$$\lambda = 4\frac{N^I - 2 \pm \sqrt{2(N^I - N^{II})(N^I + N^{II} - 4)}}{N^I + N^{II}}\omega. \tag{S119}$$

So when $\lambda < \frac{2(N^I - N^{II})}{N^I + N^{II}}\omega$ and $\lambda < 4\frac{N^I - 2 \pm \sqrt{2(N^I - N^{II})(N^I + N^{II} - 4)}}{N^I + N^{II}}\omega$, (S118) can not be found. In sum,

$$\lambda > \min\left\{\frac{2(N^I - N^{II})}{N^I + N^{II}}\omega, 4\frac{N^I - 2 - \sqrt{2(N^I - N^{II})(N^I + N^{II} - 4)}}{N^I + N^{II}}\omega\right\}. \tag{S120}$$

When $\lambda > \min\left\{\frac{2(N^I - N^{II})}{N^I + N^{II}}\omega, 4\frac{N^I - 2 - \sqrt{2(N^I - N^{II})(N^I + N^{II} - 4)}}{N^I + N^{II}}\omega\right\}$,

$$\beta \geqslant 2\frac{-\lambda\left(N^I + N^{II}\right) + 2\left(N^I - 2\right)\omega}{(N^I + N^{II})\left\{\frac{\pi}{2} + \arcsin\left[1 - \frac{2\left(N^I - N^{II}\right)}{\lambda\left(N^I + N^{II}\right)}\omega\right] + \arcsin\left[1 - \frac{2\left(N^I + N^{II} - 4\right)}{\lambda\left(N^I + N^{II}\right)}\omega\right]\right\}}. \tag{S121}$$

2) $N^I \geqslant N^{II}, \lambda - \frac{N^I + N^{II} - 4}{N^I + N^{II}}\omega \leqslant \beta < \lambda - \frac{-N^I + 3N^{II} - 4}{N^I + N^{II}}\omega$

The left endpoint $x_1 = -\frac{\beta}{\lambda} + \frac{3N^I - N^{II} - 4}{\lambda\left(N^I + N^{II}\right)}\omega$ turns (S112) into

$$y_1\left(-\frac{\beta}{\lambda} + \frac{3N^I - N^{II} - 4}{\lambda\left(N^I + N^{II}\right)}\right) = \arcsin\frac{-\beta + \frac{3N^I - N^{II} - 4}{N^I + N^{II}}\omega}{\lambda} + \arcsin\frac{-\beta + \frac{N^I + N^{II} - 4}{N^I + N^{II}}\omega}{\lambda} + \arcsin\frac{-\beta + \frac{N^I - 3N^{II} + 4}{N^I + N^{II}}\omega}{\lambda},$$
$$y_2\left(-\frac{\beta}{\lambda} + \frac{3N^I - N^{II} - 4}{\lambda\left(N^I + N^{II}\right)}\right) = \arcsin(1) + \arcsin\left[1 - \frac{2\left(N^I - N^{II}\right)}{\beta\left(N^I + N^{II}\right)}\omega\right]. \tag{S122}$$

To fulfill the first condition $y_1(x_1) \leqslant y_2(x_1)$, (S120) leads to

$$\frac{3}{\lambda}\beta^2 + \left[\frac{\pi + 2}{2} - \frac{5N^I - 3N^{II} - 4}{\lambda\left(N^I + N^{II}\right)}\omega\right]\beta - \frac{2\left(N^I - N^{II}\right)}{N^I + N^{II}}\omega \geqslant 0. \tag{S123}$$

Since $\frac{3}{\lambda} > 0$ and $\triangle \geqslant 0$ for this quadratic inequality,

$$\beta \geqslant -\frac{\pi + 2}{12}\lambda + \frac{5N^I - 3N^{II} - 4}{6\left(N^I + N^{II}\right)}\omega + \sqrt{\left[\frac{\pi + 2}{12}\lambda - \frac{5N^I - 3N^{II} - 4}{6\left(N^I + N^{II}\right)}\omega\right]^2 + \frac{2\lambda\left(N^I - N^{II}\right)}{3\left(N^I + N^{II}\right)}}. \tag{S124}$$

The right endpoint here is as same as 1) and leads to (S121) consequently.

3) $N^I \geqslant N^{II}, \beta < \lambda - \frac{N^I + N^{II} - 4}{N^I + N^{II}}\omega$

Since the left endpoint is as same as 2), we only discuss the right endpoint here. For the right endpoint $x_2 = \frac{\beta}{\lambda} + \frac{N^I + N^{II} - 4}{N^I + N^{II}}\omega$, (S112) yields

$$y_1\left(\frac{\beta}{\lambda} + \frac{N^I + N^{II} - 4}{N^I + N^{II}}\omega\right) = \arcsin\left[\frac{\beta}{\lambda} + \frac{N^I + N^{II} - 4}{\lambda\left(N^I + N^{II}\right)}\omega\right]$$
$$+ \arcsin\left[\frac{\beta}{\lambda} + \frac{-N^I + 3N^{II} - 4}{\lambda\left(N^I + N^{II}\right)}\omega\right] + \arcsin\left[\frac{\beta}{\lambda} - \frac{N^I + N^{II} - 4}{\lambda\left(N^I + N^{II}\right)}\omega\right], \tag{S125}$$
$$y_2\left(\frac{\beta}{\lambda} + \frac{N^I + N^{II} - 4}{N^I + N^{II}}\omega\right) = \arcsin\left[-1 + \frac{2\left(N^I - N^{II}\right)}{\beta\left(N^I + N^{II}\right)}\omega\right] - \frac{\pi}{2}.$$

Since $1 - \frac{2\left(N^I - N^{II}\right)}{\beta\left(N^I + N^{II}\right)}\omega \geqslant -1$ in (S123), we have

$$\beta \geqslant \frac{N^I - N^{II}}{N^{II} + N^I}\omega. \tag{S126}$$

Using the linearization, the second condition $y_1(x_2) \geqslant y_2(x_2)$ turns (S123) into

$$\frac{3}{\lambda}\beta^2 + \left[\frac{\pi + 2}{2} - \frac{N^I - 3N^{II} + 4}{\lambda\left(N^I + N^{II}\right)}\omega\right]\beta - \frac{2\left(N^I - N^{II}\right)}{N^I + N^{II}}\omega \geqslant 0. \tag{S127}$$

For this quadratic inequality (S125) with $\frac{3}{\lambda} > 0$ and $\triangle \geqslant 0$, we obtain

$$\beta \geqslant -\frac{\pi+2}{12}\lambda + \frac{N^I - 3N^{II} + 4}{6\left(N^I + N^{II}\right)}\omega + \sqrt{\left[\frac{\pi+2}{12}\lambda - \frac{N^I - 3N^{II} + 4}{6\left(N^I + N^{II}\right)}\omega\right]^2 + \frac{2\lambda\left(N^I - N^{II}\right)}{3\left(N^I + N^{II}\right)}}. \tag{S128}$$

4) $N^I < N^{II}, \beta \geqslant \lambda - \frac{N^I + N^{II} - 4}{N^I + N^{II}}\omega$

Since the left endpoint is as same as 1), the first condition $y_1(x_1) \leqslant y_2(x_1)$ also leads to (S114). It is noticed that $2\frac{N^I + 3N^{II} - 8 - 2\sqrt{(N^I + N^{II} - 4)(N^{II} - 2)}}{N^I + N^{II}}\omega \geqslant \frac{2\left(N^{II} - 2\right)}{N^I + N^{II}}\omega$ since $-2\sqrt{(N^I + N^{II} - 4)(N^{II} - 2)} \geqslant N^I + N^{II} - 4 + N^{II} - 2$. When $\lambda \geqslant 2\frac{N^I + 3N^{II} - 8 - 2\sqrt{(N^I + N^{II} - 4)(N^{II} - 2)}}{N^I + N^{II}}\omega$, there is no constraint on β for the first condition. When $\frac{2\left(N^{II} - 2\right)}{N^I + N^{II}}\omega \leqslant \lambda < 2\frac{N^I + 3N^{II} - 8 - 2\sqrt{(N^I + N^{II} - 4)(N^{II} - 2)}}{N^I + N^{II}}\omega$, (S116) is needed to satisfy the first condition. And $\lambda < \frac{2\left(N^{II} - 2\right)}{N^I + N^{II}}\omega$ is not permitted here, seen in (S113). Thus

$$\lambda > \frac{2\left(N^{II} - 2\right)}{N^I + N^{II}}\omega \tag{S129}$$

For the right endpoint $x_2 = 1 - \frac{2\left(N^{II} - N^I\right)}{\lambda\left(N^I + N^{II}\right)}\omega$, (S112) becomes

$$\begin{aligned}
y_1\left(1 - \frac{2\left(N^{II} - N^I\right)}{\lambda\left(N^I + N^{II}\right)}\omega\right) &= \frac{\pi}{2} + \arcsin\left[1 - \frac{2\left(N^{II} - N^I\right)}{\lambda\left(N^I + N^{II}\right)}\omega\right] + \arcsin\left[1 - \frac{4\left(N^{II} - 2\right)}{\lambda\left(N^I + N^{II}\right)}\omega\right], \\
y_2\left(1 - \frac{2\left(N^{II} - N^I\right)}{\lambda\left(N^I + N^{II}\right)}\omega\right) &= \arcsin\frac{-\lambda + \frac{N^I + N^{II} - 4}{N^I + N^{II}}\omega}{\beta} + \arcsin\frac{-\lambda + \frac{-N^I + 3N^{II} - 4}{N^I + N^{II}}\omega}{\beta},
\end{aligned} \tag{S130}$$

which requires $\lambda \geqslant \frac{2\left(N^{II} - 2\right)}{N^I + N^{II}}\omega$. Since $\lambda \geqslant \frac{2\left(N^{II} - 2\right)}{N^I + N^{II}}\omega$, $y_1\left(1 - \frac{2\left(N^{II} - N^I\right)}{\lambda\left(N^I + N^{II}\right)}\omega\right) > 0 \geqslant y_2\left(1 - \frac{2\left(N^{II} - N^I\right)}{\lambda\left(N^I + N^{II}\right)}\omega\right)$, indicating that the second condition is satisfied.

5) $N^I < N^{II}, \beta < \lambda - \frac{N^I + N^{II} - 4}{N^I + N^{II}}\omega$

The left endpoint $x_1 = -\frac{\beta}{\lambda} + \frac{N^I + N^{II} - 4}{\lambda\left(N^I + N^{II}\right)}\omega$ leads to

$$\begin{aligned}
y_1\left(-\frac{\beta}{\lambda} + \frac{N^I + N^{II} - 4}{\lambda\left(N^I + N^{II}\right)}\omega\right) &= \arcsin\left[-\frac{\beta}{\lambda} + \frac{N^I + N^{II} - 4}{\lambda\left(N^I + N^{II}\right)}\omega\right] \\
&+ \arcsin\left[-\frac{\beta}{\lambda} + \frac{-N^I + 3N^{II} - 4}{\lambda\left(N^I + N^{II}\right)}\omega\right] + \arcsin\left[-\frac{\beta}{\lambda} - \frac{N^I + N^{II} - 4}{\lambda\left(N^I + N^{II}\right)}\omega\right], \\
y_2\left(-\frac{\beta}{\lambda} + \frac{N^I + N^{II} - 4}{\lambda\left(N^I + N^{II}\right)}\omega\right) &= \arcsin\left[1 - \frac{2\left(N^{II} - N^I\right)}{\beta\left(N^I + N^{II}\right)}\omega\right] + \frac{\pi}{2}.
\end{aligned} \tag{S131}$$

Since $1 - \frac{2\left(N^{II}-N^{I}\right)}{\beta\left(N^{I}+N^{II}\right)}\omega \geqslant -1$, we have

$$\beta \geqslant \frac{N^{II} - N^{I}}{N^{II} + N^{I}}\omega. \tag{S132}$$

Using (S129), the linearization of $y_1(x_1) \leqslant y_2(x_1)$ yields

$$\frac{3}{\lambda}\beta^2 + \left[\frac{\pi+2}{2} + \frac{N^{I} - 3N^{II} + 4}{\lambda\left(N^{I} + N^{II}\right)}\omega\right]\beta - \frac{2\left(N^{II} - N^{I}\right)}{N^{I} + N^{II}}\omega \geqslant 0, \tag{S133}$$

leading to

$$\beta \geqslant -\frac{\pi+2}{12}\lambda - \frac{N^{I} - 3N^{II} + 4}{6\left(N^{I} + N^{II}\right)}\omega + \sqrt{\left[\frac{\pi+2}{12}\lambda + \frac{N^{I} - 3N^{II} + 4}{6\left(N^{I} + N^{II}\right)}\omega\right]^2 + \frac{2\lambda\left(N^{II} - N^{I}\right)}{3\left(N^{I} + N^{II}\right)}\omega}. \tag{S134}$$

For the right endpoint $\frac{\beta}{\lambda} + \frac{3N^{I}-N^{II}-4}{\lambda\left(N^{I}+N^{II}\right)}\omega$, (S112) becomes

$$\begin{aligned} y_1\left(\frac{\beta}{\lambda} + \frac{3N^{I} - N^{II} - 4}{\lambda\left(N^{I} + N^{II}\right)}\omega\right) &= \arcsin\left[\frac{\beta}{\lambda} + \frac{3N^{I} - N^{II} - 4}{\lambda\left(N^{I} + N^{II}\right)}\omega\right] \\ \arcsin\left[\frac{\beta}{\lambda} + \frac{N^{I} + N^{II} - 4}{\lambda\left(N^{I} + N^{II}\right)}\omega\right] &+ \arcsin\left[\frac{\beta}{\lambda} + \frac{N^{I} - 3N^{II} + 4}{\lambda\left(N^{I} + N^{II}\right)}\omega\right], \\ y_2\left(\frac{\beta}{\lambda} + \frac{3N^{I} - N^{II} - 4}{\lambda\left(N^{I} + N^{II}\right)}\omega\right) &= -\frac{\pi}{2} - \arcsin\left[1 - \frac{2\left(N^{II} - N^{I}\right)}{\beta\left(N^{I} + N^{II}\right)}\omega\right]. \end{aligned} \tag{S135}$$

The linearization of the second condition $y_1\left(\frac{\beta}{\lambda} + \frac{3N^{I}-N^{II}-4}{\lambda\left(N^{I}+N^{II}\right)}\omega\right) \geqslant y_2\left(\frac{\beta}{\lambda} + \frac{3N^{I}-N^{II}-4}{\lambda\left(N^{I}+N^{II}\right)}\omega\right)$ leads to

$$\frac{3}{\lambda}\beta^2 + \left[\frac{\pi+2}{2} + \frac{5N^{I} - 3N^{II} - 4}{\lambda\left(N^{I} + N^{II}\right)}\omega\right]\beta - \frac{2\left(N^{II} - N^{I}\right)}{N^{I} + N^{II}}\omega \geqslant 0, \tag{S136}$$

which means

$$\beta \geqslant -\frac{\pi+2}{12}\lambda - \frac{5N^{I} - 3N^{II} - 4}{6\left(N^{I} + N^{II}\right)}\omega + \sqrt{\left[\frac{\pi+2}{12}\lambda + \frac{5N^{I} - 3N^{II} - 4}{6\left(N^{I} + N^{II}\right)}\omega\right]^2 + \frac{2\lambda\left(N^{II} - N^{I}\right)}{3\left(N^{I} + N^{II}\right)}\omega}. \tag{S137}$$

In sum,

$$\lambda_c^{\textbf{H-L L-L}} = \frac{N^{I} + N^{II} - 4}{N^{I} + N^{II}}\omega, \tag{S138}$$

$$\beta_{min}^{\textbf{H-L L-L}} = \frac{\left|N^{I} - N^{II}\right|}{N^{I} + N^{II}}\omega. \tag{S139}$$

For $N^I \geqslant N^{II}$,

$$\beta_c^{\textbf{H-L L-L}} = \max\begin{cases} 2\dfrac{-\lambda\left(N^I+N^{II}\right)+2\left(N^I-2\right)\omega}{\left(N^I+N^{II}\right)\left\{\frac{\pi}{2}+\arcsin\left[1-\frac{2\left(N^I-N^{II}\right)}{\lambda\left(N^I+N^{II}\right)}\omega\right]+\arcsin\left[1-\frac{2\left(N^I+N^{II}-4\right)}{\lambda\left(N^I+N^{II}\right)}\omega\right]\right\}}, \lambda < \dfrac{2\left(N^I-2\right)}{N^I+N^{II}}\omega \\ -\dfrac{\pi+2}{12}\lambda+\dfrac{5N^I-3N^{II}-4}{6\left(N^I+N^{II}\right)}\omega+\sqrt{\left[\dfrac{\pi+2}{12}\lambda-\dfrac{5N^I-3N^{II}-4}{6\left(N^I+N^{II}\right)}\omega\right]^2+\dfrac{2\lambda\left(N^I-N^{II}\right)}{3\left(N^I+N^{II}\right)}} \end{cases}, \tag{S140}$$

For $N^I < N^{II}$,

$$\beta_c^{\textbf{H-L L-L}} = -\frac{\pi+2}{12}\lambda-\frac{N^I-3N^{II}+4}{6\left(N^I+N^{II}\right)}\omega+\sqrt{\left[\frac{\pi+2}{12}\lambda+\frac{N^I-3N^{II}+4}{6\left(N^I+N^{II}\right)}\omega\right]^2+\frac{2\lambda\left(N^{II}-N^I\right)}{3\left(N^I+N^{II}\right)}}, \tag{S141}$$

Moreover, the maximal external coupling strength exits:

- for $\lambda < 2\frac{N^I+3N^{II}-8-2\sqrt{(N^I+N^{II}-4)(N^{II}-2)}}{N^I+N^{II}}\omega$

$$\beta_{max}^{\textbf{H-L L-L}} = 2\frac{\lambda\left(N^I+N^{II}\right)-2\left(N^{II}-2\right)\omega}{\left(N^I+N^{II}\right)\left\{\arcsin\left[\frac{2\left(N^I+N^{II}-4\right)}{\lambda\left(N^I+N^{II}\right)}\omega-1\right]+\arcsin\left[\frac{4\left(N^{II}-2\right)}{\lambda\left(N^I+N^{II}\right)}\omega-1\right]-\frac{\pi}{2}\right\}}; \tag{S142}$$

- for $\lambda < 4\frac{N^I-2-\sqrt{2(N^I-N^{II})(N^I+N^{II}-4)}}{N^I+N^{II}}\omega$

$$\beta_{max}^{\textbf{H-L L-L}} = 2\frac{\lambda\left(N^I+N^{II}\right)-2\left(N^I-2\right)\omega}{\left(N^I+N^{II}\right)\left\{\arcsin\left[\frac{2\left(N^I-N^{II}\right)}{\lambda\left(N^I+N^{II}\right)}\omega-1\right]+\arcsin\left[\frac{2\left(N^I+N^{II}-4\right)}{\lambda\left(N^I+N^{II}\right)}\omega-1\right]\right\}-\frac{\pi}{2}} \tag{S143}$$

In two-links connection, we derive the critical values for two kinds of coupling strengths, λ and β, the validations about which are shown in Fig. S4 and Fig. S5 respectively. In Fig. S4 and Fig. S5, there are 8 sub-figures that separate into two columns: 1) the first column includes **a**, **c**, **e** and **g**, describing the non-synchronized phase; 2) the second column includes **b**, **d**, **f** and **h**, capturing the synchronized state. There are four rows representing four cases respectively. For example, sub-figures **a** and **b** in the first row are for the same **H-H L-L** case.

In Fig. S4, all the plots are about the simulation with $N^I = 16, N^{II} = 11, \omega = 1, \beta = 10$. But the first column is with $\lambda = \lambda_c - 0.01$, while the second column is with $\lambda = \lambda_c + 0.01$. λ_c found in the simulations for **H-H L-L**, **H-L L-H**, **L-L L-L** and **H-L L-L** are 0.86, 0.86, 1.04 and 1.09 respectively. The first three values are in consistent with the values derived from (S46), (S72) and (S94), for . As to **H-L L-L** case, it is noticed that $\beta = 10$ is a very large value and there exits the upper limit for β when $\lambda \leqslant \frac{(N^I+3N^{II}-8)-2\sqrt{(N^I+N^{II}-4)(N^{II}-2)}}{N^I+N^{II}}\omega$, seen in (S116). So the system can be

synchronized if $\lambda \geqslant 2\frac{(N^I+3N^{II}-8)-2\sqrt{(N^I+N^{II}-4)(N^{II}-2)}}{N^I+N^{II}}\omega = 1.11$, which is close to 1.10 found in the simulation.

In Fig. S5, we ran the simulation with $N^I = 16, N^{II} = 11, \omega = 1, \lambda = 10$ for all plots. There are two columns: the first column is about $\beta_c - 0.01$; the second column is about $\beta_c + 0.01$. For all the connection modes in two-links connection, β_c found in the simulations are 0.19, which are in consistent with the values derived from (S48), (S74), (S95) and (S137).

In Figs. S4 and S5, the values derived are very close to the values obtained from the simulations, which work as the verification.

In all connection modes, the values of $\sin a$ are the same. Since $\sin a$ dominates the order parameter, the synchronization levels for different connection modes are nearly the same.

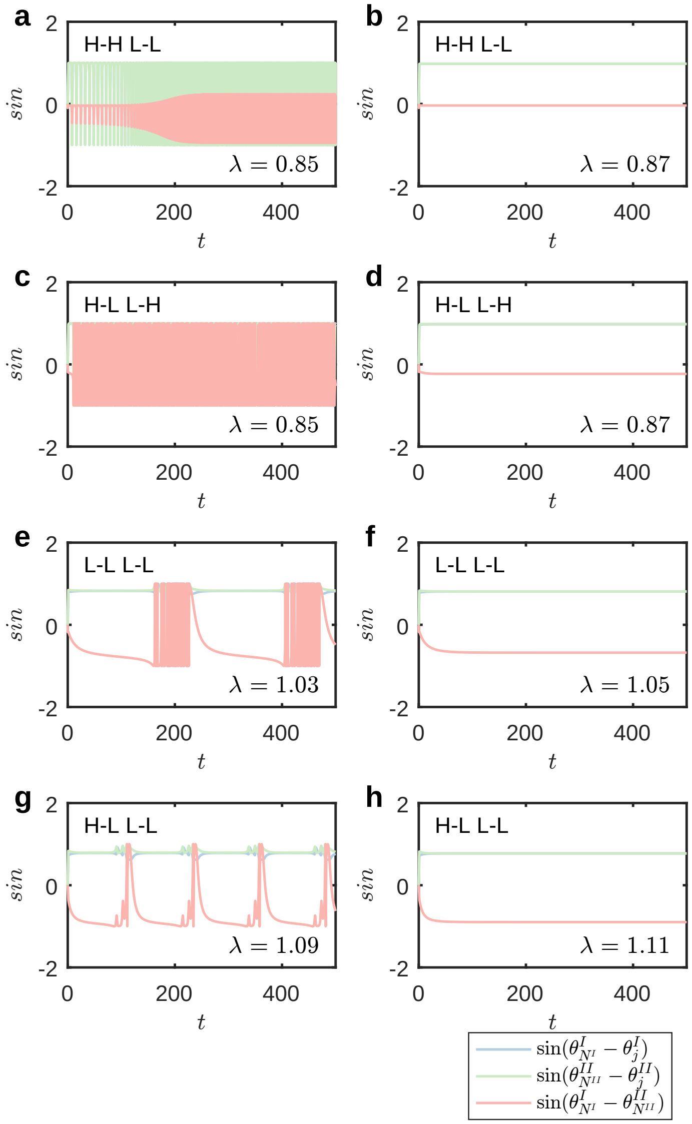

Figure S4: The critical internal coupling strength in two-links connection. We ran Kuramoto model (S2) on two coupled networks with $N^I = 16, N^{II} = 11, \omega = 1, \beta = 10$, for different λ. Phase differences are shown, including the one between the hub and the leaves (not the connector, blue curve for Network I and red curve for Network II) as well as the one between the hub and the connector (green curve for Network I and light blue curve for Network II). Case **H-H L-L**: $\lambda = 0.85$ in **a**; $\lambda = 0.87$ in **b**. Case **H-L L-H**: $\lambda = 0.85$ in **c**; $\lambda = 0.87$ in **d**. Case **L-L L-L**: $\lambda = 1.03$ in **e**; $\lambda = 1.05$ in **f**. Case **H-L L-L**: $\lambda = 1.09$ in **g**; $\lambda = 1.11$ in **h**.

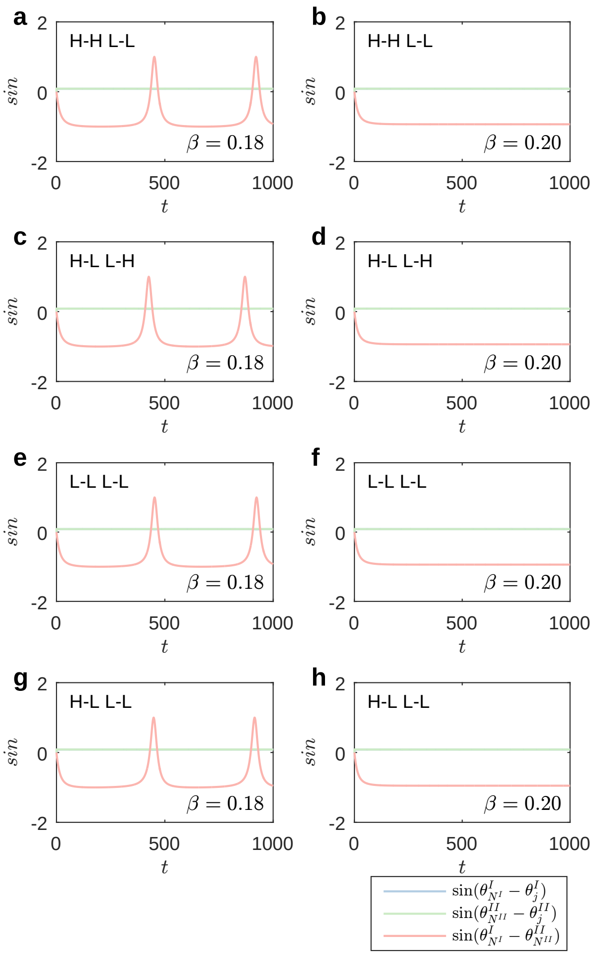

Figure S5: The critical external coupling strength in two-links connection. We ran Kuramoto model (S2) on two coupled networks with $N^I = 16, N^{II} = 11, \omega = 1, \lambda = 10$, for different β. Phase differences are shown, including the one between the hub and the leaves (except the leaf connector, blue curve for Network I and red curve for Network II) as well as the one between the hub and the connector (green curve for Network I and light blue curve for Network II). Case **H-H L-L**: $\beta = 0.18$ in **a**; $\beta = 0.20$ in **b**. Case **H-L L-H**: $\beta = 0.18$ in **c**; $\beta = 0.20$ in **d**. Case **L-L L-L**: $\beta = 0.18$ in **e**; $\beta = 0.20$ in **f**. **H-L L-L**: $\beta = 0.18$ in **g**; $\beta = 0.20$ in **h**.

S4 A special case: the two network sizes are equal

According to the result derived in S2 and S3, $\beta_c = 0$ and λ_c are the same for all connection modes when $N^I = N^{II}$. This special case is validated through the simulations using (S2) with $N^I = N^{II} = 20, \omega = 1$, shown in Table. S4. It can be seen that for all the connection modes, even if β is as small as 0.01, the system can still be synchronized when λ is larger than the critical value. This result indicates that two coupled networks seem to be uncoupled when their network sizes are equal.

For **H-L L-L** case with $\lambda = 0.91$, there exits β_{max}, which equals to 0.04 according to (S116). Thus, the two networks coupled through **H-L L-L** connection mode with $\lambda = 0.91, \beta = 0.10$ cannot be synchronized.

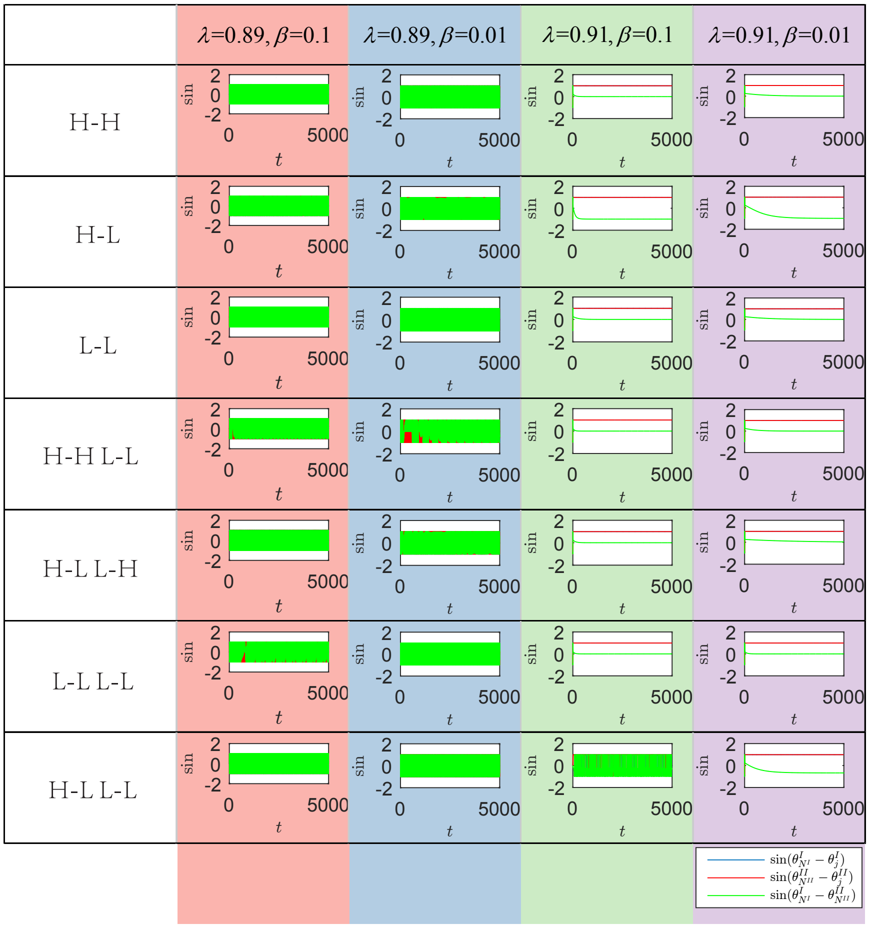

Table S4: The equal network sizes. The Kuramoto dynamics (S2) was cast on two networks with $N^I = N^{II} = 20, \omega = 1$. Phase difference between the hub and the leaves (the leaves are not the connectors, blue curve for Network I and red curve for Network II) and the one between two hubs (green curve) are given for coupling strengths (1) $\lambda = 0.89, \beta = 0.01$ (red), (2) $\lambda = 0.89, \beta = 0.10$ (blue), (3) $\lambda = 0.91, \beta = 0.01$ (green) and (4) $\lambda = 0.91, \beta = 0.01$ (purple).

S5 Different roles between internal and external coupling strengths

To understand the differences between λ and β, synchronization areas with three groups of coupling strengths are compared:

- Group 1: $\lambda = 0.99, \beta = 1.00$ (Fig. S6);
- Group 2: $\lambda = 0.99, \beta = 0.50$ (Fig. S7);
- Group 3: $\lambda = 0.98, \beta = 1.00$ (Fig. 4**a-g** in the manuscript);

Here, we define *size* and *shape* for discussion conveniently as follows:

- *size*: in the synchronization area of case **H-H**, the max value along the diagonal line $N^{II} = N^{I}$.
  For Groups 1 and 2, *size* = 200.
  For Group 3, *size* = 100.
- *shape*: in the synchronization area of case **H-H**, the approximate appearance of the boundary.
  For Groups 1 and 3, the *shape* of their synchronization areas is **a equilateral triangle**.
  For Group 2, the *shape* of its synchronization area is **a skinny triangle**.

In one hand, Group 1 and Group 2 share the same λ and *size*, while they have different β and *shape*. In the other hand, Group 1 and Group 3 have different λ and *size*, while they share the same β and *shape*. Thus, we can draw a conclusion that λ controls the *size* of the synchronization area while β decides its *shape*.

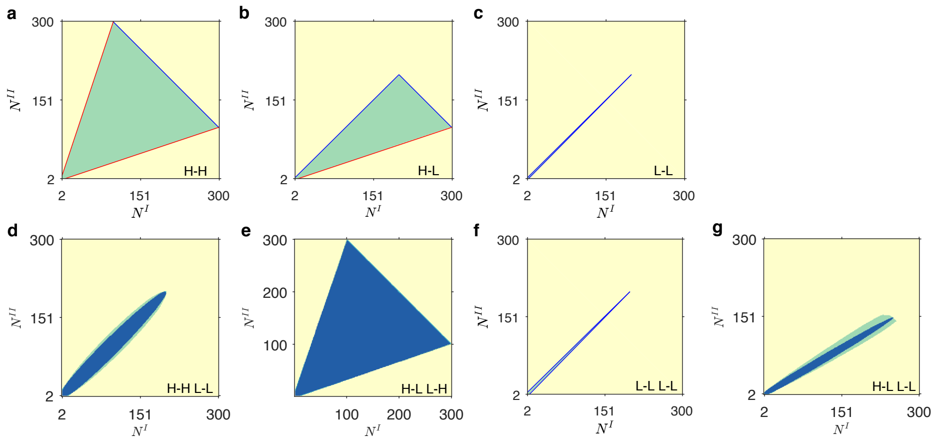

Figure S6: Phase diagram with $\lambda = 0.99, \beta = 1.00$. In **a**, **b**, **c** and **f**, the simulation result is shown by the green zone (the synchronization area) and the yellow zone (the non-synchronization area), while the theoretical result is indicated through red curves ($\beta_c = 1.00$ for (S11), (S21), (S28) and (S95) respectively) and blue curves ($\lambda_c = 0.99$ for (S10), (S20), (S27) and (S94) respectively). In **d**, **e** and **g**: presents the non-synchronization are obtained through the theory and the simulation; stands for the synchronization area obtained by the theory and the simulation; shows the synchronization area obtained only by simulation; illustrates the synchronization area obtained only by simulation.

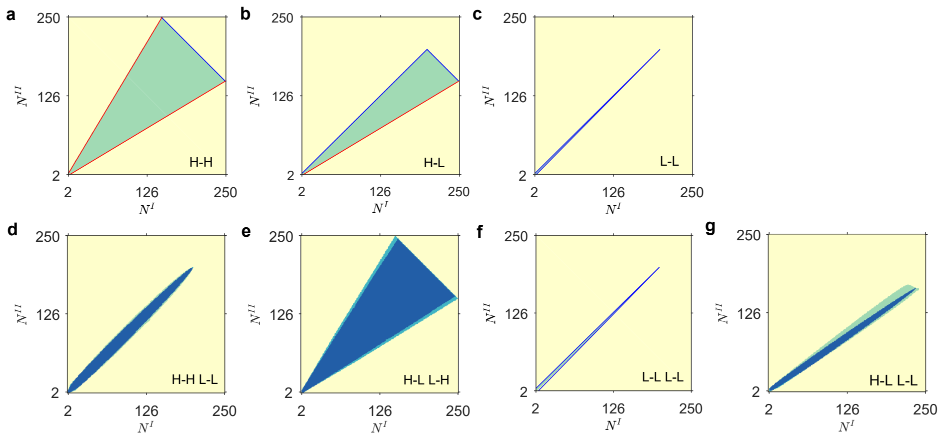

Figure S7: Phase diagram with $\lambda = 0.99, \beta = 0.50$. In **a**, **b**, **c** and **f**, the simulation result is shown by the green zone (the synchronization area) and the yellow zone (the non-synchronization area), while the theoretical result is indicated through red curves ($\beta_c = 0.50$ for (S11), (S21), (S28) and (S95) respectively) and blue curves ($\lambda_c = 0.99$ for (S10), (S20), (S27) and (S94) respectively). In **d**, **e** and **g**: presents the non-synchronization are obtained through the theory and the simulation; stands for the synchronization area obtained by the theory and the simulation; shows the synchronization area obtained only by simulation; illustrates the synchronization area obtained only by simulation.

S6 Phase diagram composed of hub degrees

Previous studies[1,6] regard the node number in a star network equals to the hub degree in a SF network. To be specific, the result derived through a star network with the node number N will be validated in a SF network with the hub degree K_H. We also use such validation, the result of which is shown in Fig. S8. In Fig. S8, phase diagram of SF networks is the result of five groups of simulations, based on five pairs of SF networks respectively. The setting of five pairs is because the random generation of SF networks. For one pair of SF networks, the degrees of their hubs are K^I and K^{II} respectively. The more pairs of SF networks with K^I and K^{II} are synchronized under the condition $\lambda = 0.99, \beta = 1.00, \omega = 1$, the darker color of corresponding dot (K^I, K^{II}) in Fig. S8. Such dots constitute the synchronization area of SF networks. Meanwhile, the synchronization area of star networks is shown by red curves, which capture the boundary of the synchronization area. As pointed out in Fig. S8, the validation composed of hub degrees is not ideal since the synchronization areas between SF networks and star networks are very different.

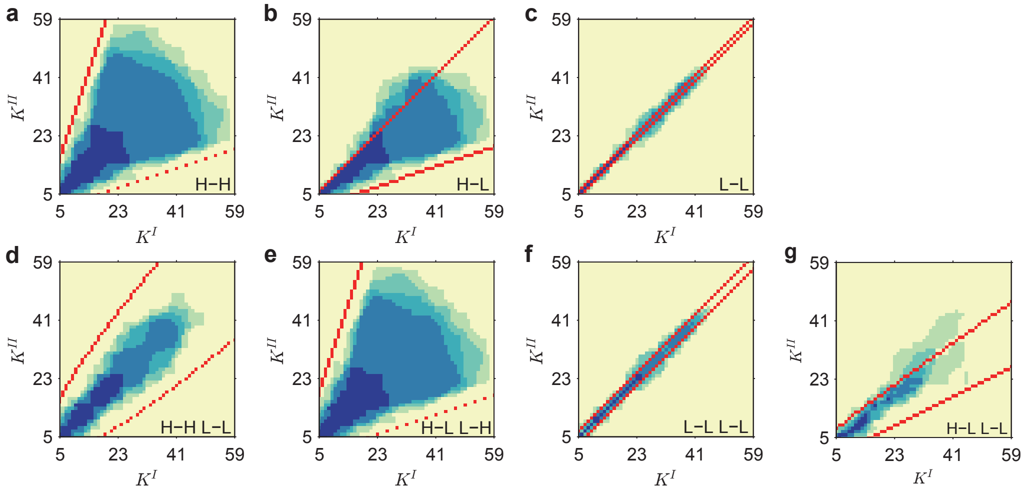

Figure S8: Phase diagram based on combinations of K^I and K^{II}. There are five pairs of two coupled SF networks in all plots. More pairs with K^I and K^{II} are synchronized through the simulations of (S2) with $\lambda = 0.99, \beta = 1.00, \omega = 1$, the color of corresponding dot(K^I, K^{II}) in all plots is darker. The red curve is the boundary of the synchronization area of star networks under the same conditions (Fig. S6). The results for all six connection modes are shown: **a** for **H-H**; **b** for **H-L**; **c** for **L-L**; **d** for **H-H L-L**; **e** for **H-L L-H**; **f** for **L-L L-L**; **g** for **H-L L-L**.

Reference

1. Gómez-Gardeñes, J., Gómez, S., Arenas, A. & Moreno, Y. Explosive synchronization transitions in scale-free networks. *Physical Review Letters* **106**, 128701 (2011).

2. Dörfler, F. & Bullo, F. Kron reduction of graphs with applications to electrical networks. *Circuits and Systems I: Regular Papers, IEEE Transactions on* **60**, 150–163 (2011).

3. Simpson-Porco, J., Dörfler, F. & Bullo, F. Synchronization and power sharing for droop-controlled inverters in islanded microgrids. *Automatica* **49**, 2603–2611 (2013).

4. Aguirre, J., Papo, D. & Buldú, J. Successful strategies for competing networks. *Nature Physics* **9**, 230–234 (2013).

5. Aguirre, J., Sevilla-Escoboza, R., Gutiérrez, R., Papo, D. & Buldú, J.M. Synchronization of interconnected networks: the role of connector nodes. *Physical review letters* **112**, 248701 (2014).

6. Zou, Y., Pereira, T., Small, M., Liu, Z. & Kurths, J. Basin of attraction determines hysteresis in explosive synchronization. *Physical review letters* **112**, 114102 (2014).

7. Kundur, P. *Power System Stability and Control* (McGraw-Hill Professional, 2005).
